# Deep learning approaches for resolving genomic discrepancies in cancer: a systematic review and clinical perspective

**DOI:** 10.1093/bib/bbaf541

**Published:** 2025-11-02

**Authors:** Muhammad Zubair, Ali Haider Khan, Syed Fakhar Bilal, Jianqiang Li

**Affiliations:** Faculty of Information Technology, Beijing University of Technology, Beijing 100124, China; Faculty of Information Technology, Beijing University of Technology, Beijing 100124, China; Faculty of Information Technology, Beijing University of Technology, Beijing 100124, China; Faculty of Information Technology, Beijing University of Technology, Beijing 100124, China

**Keywords:** deep learning, cancer genomics, genomic discrepancies, mutation detection, variant calling, whole exome sequencing (WES), multi-omics integration, clinical bioinformatics

## Abstract

Discrepancies in cancer sequencing data continue to pose significant challenges for accurate mutation detection, potentially resulting in misdiagnoses and suboptimal treatment strategies. Although deep learning (DL) has emerged as a transformative approach for identifying and rectifying these errors, there remains a lack of comprehensive evaluation of DL architectures, performance benchmarks, and clinical translation. In this systematic review of 78 studies (2015–2024), We synthesize recent advancements in DL methodologies for identifying genomic discrepancies, demonstrating that convolutional and graph-based architectures currently achieve state-of-the-art performance in variant calling and tumor stratification. DL models reduce false-negative rates by 30%–40% compared to traditional pipelines, with methods such as MAGPIE prioritizing pathogenic variants with 92% accuracy. However, challenges such as data scarcity, batch effects, and the interpretability of “black-box” models persist. We propose a future research roadmap advocating federated learning to enhance data privacy and attention mechanisms to improve model transparency. By bridging bioinformatics and oncology, this review offers actionable insights to expedite the deployment of DL in precision cancer therapy.

## Introduction

Cancer continues to pose a significant global health challenge, accounting for over 10 million deaths annually and ranking as the second leading cause of mortality worldwide, following cardiovascular diseases (WHO 2020; GLOBOCAN 2020) [1, 2]. The rising global incidence and mortality rates of cancer highlight the urgent need for more effective diagnostic and therapeutic strategies [2, 3]. Advances in genomic technologies, particularly next-generation sequencing (NGS) and whole-exome sequencing (WES), have transformed our capacity to profile the cancer genome, identify driver mutations, and develop targeted therapies [4, 5]. However, the accuracy of mutation detection is often constrained by persistent discrepancies in cancer genomic data, including sequencing artifacts, coverage biases, and variant-calling errors [6, 7]. These discrepancies can lead to false-positive or false-negative findings, resulting in misdiagnosis, inappropriate treatment selection, and ultimately, poorer patient outcomes [8–10]. The integration of multi-omics data, while promising for deeper biological insights, further complicates challenges due to batch effects and data harmonization difficulties [11, 12].

Traditional bioinformatics pipelines frequently struggle to keep pace with the volume and complexity of modern cancer datasets and are limited in their ability to recognize subtle or nonlinear patterns [13]. In recent years, artificial intelligence (AI), particularly DL, has emerged as a transformative approach in cancer genomics, enabling scalable, high-precision analyses that surpass conventional methods [14–16]. DL architectures, such as convolutional neural networks (CNNs) and graph neural networks (GCNs), have achieved state-of-the-art performance in detecting mutations, classifying variants, and subtyping tumors [17–19]. Convolutional and graph-based DL models now achieve state-of-the-art accuracy in variant detection and enable more informative multi-omics tumor subtyping and biomarker discovery (see Section 3 and Table 1).

**Table 1:** Summary of deep learning models for resolving genomic discrepancies in cancer. Key representative deep learning models are presented with their core architectures, data types, primary datasets, benchmark performance metrics, main applications in cancer genomics, and relevant references. This table underscores the diversity of approaches, ranging from CNNs for variant calling to multimodal models for survival prediction, and their impact on precision oncology.

**Model name**	**Architecture**	**Data type(s)**	**Dataset(s)**	**Metric (value; type)**	**Main application**	**Key advantages**	**Ref**
DeepVariant	CNN	WGS, WES	GIAB, TCGA	99.1% (SNV accuracy)	Germline/Somatic Variant Calling	Learns read-level error context; reduces INDEL false positives	[18]
NeuSomatic	CNN	WGS, WES (tumor/normal)	DREAM, in-silico spike-ins	~98% precision; 40% INDEL FPs	Somatic Variant Calling	Synthetic-data training; robust to caller disagreement	[36]
DeepGene	DNN	Somatic mutations	TCGA	+24% over SVM (F1-score)	Tumor Type Classification	Handles sparse mutation vectors; improves class balance	[54]
PathDSP	Pathway-aware DL	Expression ± CNA	GDSC/CCLE	drug-response AUROC (report value used)	Drug Sensitivity Prediction	Uses biological pathways → interpretability	[96]
DeepCNA	CNN (expression+CNA)	Copy number + RNA-seq	TCGA (multi-cancer)	Reported accuracy/AUC (insert)	Cancer Type Classification	Joint modeling of CNA & expression	[78]
MAGPIE	Attention multimodal NN	WES + transcriptome + phenotype	Rare disease cohorts	92% (variant prioritization accuracy)	Variant Prioritization (VUS)	Attention over modalities; patient-level phenotypes	[47]
Pathomic Fusion	Multimodal (CNN + GNN/GCN)	Histology + genomics/CNV	TCGA + private	0.89 (C-index, survival) versus 0.79 genomics	Survival Prediction	Fuses image & omics; weights heterogeneous inputs	[44]
Coudray *et al.*	CNN (Inception/ResNet)	Histopathology WSIs	TCGA NSCLC	94% (subtype accuracy)	Tumor Subtyping	End-to-end slide analysis; mutation inference	[23]

Despite these advances, the clinical translation of DL in cancer genomics is impeded by unresolved challenges, including sequencing discrepancies, data scarcity, limited model interpretability, and barriers to adoption in healthcare settings [7, 21, 22]. A systematic and critical evaluation of existing DL methods is essential to guide future research and clinical implementation. This systematic review addresses these needs by critically synthesizing recent advances in DL for detecting and resolving genomic discrepancies in cancer. We assessed the strengths, limitations, and translational potential of current DL approaches, aiming to provide actionable insights for both researchers and clinicians and to chart a future roadmap for AI-driven precision cancer medicine.

### Systematic structure of the review

Despite significant advancements in sequencing technologies, genomic data in cancer remain vulnerable to discrepancies, including sequencing errors, variant misclassification, and integration noise [4–7]. For instance, even clinical-grade WES exhibits false-negative rates of 5%–10% for single-nucleotide variants (SNVs) and up to 15%–20% for insertions and deletions (INDELs), primarily because of coverage biases and algorithmic limitations in conventional pipelines [6, 8]. Notably, analyses of large consortia, such as The Cancer Genome Atlas (TCGA), indicate that a substantial fraction of pathogenic mutations may be overlooked in WES datasets because of factors such as low tumor purity or insufficient sequencing depth [9, 10]. These inaccuracies have tangible clinical implications; e.g. studies have demonstrated that the reassessment of cancer mutations using DL approaches can alter clinical management and improve outcomes [14, 16]. Traditional variant calling and annotation pipelines often encounter challenges with the scale, noise, and heterogeneity of modern cancer datasets [13]. In contrast, DL architectures, including CNNs and transformer-based models, facilitate automated feature extraction and have shown substantial improvements, reducing false-negative rates by 30%–40% in somatic variant detection [14, 16, 17]. Similarly, integrated DL frameworks that leverage phenotype and genotype data, such as MAGPIE and other models, have enhanced the prioritization of disease-causing variants compared with traditional tools [13, 18]. While these advances underscore DL’s transformative potential of DL for resolving genomic discrepancies, key challenges persist, including limited reproducibility, lack of clinical validation, and incomplete integration of multi-omics data [7, 19, 20].

This systematic review critically evaluates the extent to which major DL architectures, including CNNs, recurrent neural networks (RNNs), and graph convolutional networks (GCNs), address discrepancies in cancer genomic data through automated noise reduction, multimodal data integration, and improved variant prioritization. We highlight the clinical successes, persistent challenges, and future directions for deploying DL in precision oncology. The core sections of this review are as follows.

- Types of genomic data for analyzing discrepancies: overview of sequencing technologies, such as WES, NGS, and WGS, and their respective roles in mutation detection and cancer genomics [4, 5].
- DL architectures for genomic discrepancies: examination of DL models, including CNNs, RNNs, and long short-term memory networks (LSTMs), and how they outperform traditional methods in uncovering hidden patterns in genomic data [14, 16–19].
- Impact of discrepancies on mutation identification and classification: analysis of how sequencing and data integration errors affect the identification, classification, and downstream management of cancer mutations [6–8].
- Research methodology: assessment of review methods, limitations, and the importance of multi-omics data integration for a comprehensive understanding of cancer genomics [11, 12].
- Future trends: discussion of the remaining challenges in genomic data handling and emerging DL-based approaches to address these obstacles [20–22].

## Types of genomic data for discrepancy analysis

Genomic data, the fundamental blueprint encoded in DNA, govern biological processes and disease susceptibility. Recent advances in sequencing have enabled large-scale, high-resolution analyses of cancer genomes, revolutionizing our understanding of oncogenesis and molecular pathology.

### Sequencing strategies: WES and WGS

WES and WGS are complementary NGS methodologies distinguished primarily by their scope, cost, and processing time. WES focuses on ~1% of the genome i.e. protein-coding, offering a more expedient and cost-efficient approach for identifying clinically significant variants in oncology and rare disease diagnostics [23, 24]. In contrast, WGS examines the entire genome, encompassing SNVs, INDELS, structural and copy-number alterations, and noncoding or epigenetic modifications, thereby providing the most exhaustive molecular profile for research and advanced precision oncology applications [26]. Despite a reduction in WGS costs of over 90% since 2015, many institutions continue to favor WES because of its reduced sequencing and analysis demands and quicker turnaround [24]. In practice, WES is employed for the rapid prioritization of variants in established cancer genes, whereas WGS is selected when noncoding drivers, complex rearrangements, or genome-wide mutational signatures are critical. Consequently, downstream DL pipelines must consider the distinct coverage, noise characteristics, and variant spectra inherent to these two data sources.

### Next-generation sequencing

NGS enables rapid, parallel sequencing of entire genomes or targeted regions, transforming cancer research by revealing the full spectrum of somatic mutations and tumor heterogeneity [25]. This technology captures both coding and noncoding DNA regions, revealing the complex interactions and genomic alterations that drive cancer development and progression.

### Key genomic datasets

The utility of WES, NGS, and WGS is amplified by the availability of large and high-quality genomic datasets. Landmark resources such as TCGA [27], the Catalogue of Somatic Mutations in Cancer (COSMIC) [28], Cancer Cell Line Encyclopedia (CCLE) [29], the 1000 Genomes Project [30], Pan-Cancer Analysis of Whole Genomes (PCAWG) [31], and Gene Expression Omnibus (GEO) [32] have accelerated discoveries in cancer genomics. TCGA datasets often exclude samples with low coverage or tumor purity to improve the reliability of the data. Multi-omics harmonization, such as the normalization of RNA-seq and methylation data using tools such as ComBat, helps correct batch effects across sequencing platforms and populations [33]. These diverse, harmonized datasets spanning multiple cancer types and populations are critical for training and evaluating DL models for detecting and classifying mutations.

### Understanding discrepancies in genomic data

Discrepancies in genomic data encompass inconsistencies and errors that arise during sequencing, processing and integration. Sequencing errors, such as incorrect nucleotide incorporation or low-quality base calls, can distort the identification of mutations [34]. The integration of datasets from heterogeneous sources introduces batch effects and technical variability, complicating downstream analysis [33]. Quality control and recalibration methods, such as GATK’s base quality score recalibration (BQSR) of GATK, are employed to mitigate these artifacts. DL-based methods, such as NeuSomatic, trained on synthetic benchmark datasets, have demonstrated a 40% reduction in INDEL false positives compared to classical pipelines, achieving precision rates of up to 98.5% [35].

These discrepancies have profound biological and clinical implications. Errors or inconsistencies can obscure pathogenic variants, delaying diagnosis or misguiding treatment. For instance, undetected mutations or false negatives may prevent timely intervention in cancer care, whereas batch effects can undermine the reproducibility of research findings [33, 36]. Integrated DL models, such as Pathomic Fusion, combine histological images with genomic data to predict cancer outcomes more accurately by leveraging multimodal information and attention mechanisms to control data heterogeneity [37]. These approaches illustrate the potential for resolving contradictions in genomic data and advancing precision oncology. Ultimately, minimizing discrepancies is essential to ensure robust variant interpretation, reliable biomarker discovery, and improved patient care [38].

Genomic discrepancies originate from two primary sources: (i) technical artifacts introduced during sequencing, alignment, and data harmonization, and (ii) genuine biological complexity that challenges deterministic calling rules. Base-calling errors, PCR/amplification bias, and mapping ambiguities can result in false SNVs/INDELs or inaccurately estimated allele fractions [35, 57]. The integration of data from heterogeneous platforms or centers further introduces batch effects and technical variability, which distort downstream analyses [13, 34].

Standard pipelines employ quality control and recalibration, such as GATK’s BQSR and hard-filtering heuristics, to mitigate these errors [7, 35]. DL enhances these processes. CNN-based callers (e.g. NeuSomatic) trained on synthetic and spike-in benchmarks reduce INDEL false positives by ~40% and achieve a precision near 98% on held-out sets [36]. Denoising autoencoders and variational autoencoders (VAEs) compress noisy, high-dimensional profiles to reduce spurious signals [73, 76, 95]. GANs generate realistic minority-class variants to address imbalances [62, 68]. Tumor heterogeneity, subclonal evolution, copy number alterations, structural variants, and epigenetic or RNA-editing events create realbut challenging to disentangle differences across samples and time points [10, 11, 32]. These phenomena can obscure pathogenic variants (false negatives) or exaggerate the apparent discordance between assays. GCNs and pathway-aware models integrate multi-omic and network contexts to uncover subtype-specific modules and resolve conflicts that single-omic views overlook [51, 52, 96]. Multimodal frameworks, such as Pathomic Fusion, further combine histology with genomic features to enhance outcome prediction by explicitly weighting heterogeneous inputs (see Section 4.2) [44]. Collectively, minimizing both technical and biological discrepancies is crucial for robust variant interpretation, reproducible biomarker discovery, and clinically actionable decision making [38].

## Deep learning architectures for genomic discrepancies

DL architectures have significantly transformed the resolution of discrepancies in cancer genomic data by automating feature extraction, enhancing mutation classification, and integrating multi-omics insights for more robust variant interpretation [39, 40]. In contrast to traditional methods, which often struggle with the volume and complexity of contemporary cancer genomics, DL models uncover subtle patterns and rectify inconsistencies, thereby improving accuracy and efficiency in both research and clinical practice. [41].

For instance, frameworks that integrate somatic mutation profiles with advanced classifiers, such as random forest and neural networks, have achieved over 88% accuracy in determining tissue-of-origin across various cancer types, particularly when normalization and cross-validation strategies are employed [42]. DL models, especially CNNs, facilitate the direct analysis of raw sequencing reads or genomic sequences, surpassing classical approaches in detecting pathogenic mutations and subtle sequence patterns [43].

The integration of multi-omics data with DL further propels the field by combining genomic, transcriptomic, and proteomic information to elucidate the complex molecular interactions and regulatory mechanisms underlying cancer [44, 45]. Although multi-omics integration presents challenges of high dimensionality and data variability, DL models, when appropriately standardized and made transparent, offer reproducible solutions for precision oncology [46].

Various DL models have been specifically adapted to address the challenges of genomic data analysis.

- CNNs are particularly effective for recognizing spatial patterns, such as variant calling from sequencing reads and classifying tumor subtypes from histopathological images.
- RNNs and LSTM networks are adept at modeling sequential dependencies in DNA/RNA, which is crucial for variant effect prediction and splicing analyses.
- GCNs are proficient in modeling complex biological networks and integrating multi-omics data, thereby uncovering patterns that are not accessible using other methods.
- Autoencoders, including VAEs, offer unsupervised methods for anomaly detection and dimensionality reduction in high-dimensional genomic data sets.

Recent advancements in this field include the development of PathDSP and related models for predicting drug responses using pathway-level data. In addition, DeepGene employs sparsity reduction and gene clustering techniques to enhance the classification based on somatic mutations. MAGPIE is a multimodal framework that integrates genomic, transcriptomic, and phenotypic data using attention mechanisms to prioritize pathogenic variants [54]. For instance, DeepVariant, a CNN-based variant caller, demonstrates a 99.1% accuracy rate on Genome in a Bottle benchmarks and reduces INDEL errors by 40% compared with traditional pipelines [43]. Furthermore, MAGPIE achieves a 92% accuracy rate in prioritizing disease-causing variants within rare disease cohorts, thereby reducing diagnostic odysseys by 40% [47].

DL architectures have advanced to automate feature extraction, reconcile conflicting variant calls, and integrate heterogeneous omics data, thereby enhancing the consistency and clinical relevance of cancer genomics analyses [39–41]. In comparison to traditional pipelines, DL reveals subtle patterns in sequencing reads and multi-omics networks, thus improving the accuracy of tissue-of-origin prediction, variant effect classification, and survival modeling [42, 44–46]. Beyond a single dominant architecture, various DL families have been adapted for genomic data: CNNs capture spatial patterns in read pileups and images; RNNs/LSTMs and related sequence models learn nucleotide-level dependencies; GCNs/GNNs exploit biological graphs for multi-omics integration; and autoencoders/VAEs compress high-dimensional omics for denoising and anomaly detection. Their typical strengths and limitations, such as computational cost and interpretability, are summarized in Table 2 and detailed in Sections 3.1–3.5.

**Table 2:** Limitations of computational models in genomic data analysis. Each model presents unique challenges, including computational demands, interpretability, scalability, and data integration barriers, emphasizing the need for context-appropriate model designs in precision oncology.

**Model type**	**Key limitations**	**Proposed solutions / mitigation strategies**
AI & NGS Integration	High computational requirements, need for expert bioinformatics support, privacy concerns, data interpretation challenges	Implement privacy-preserving federated learning to enable collaboration among centers without the need to transfer raw genomic data. Containerized pipelines, such as Nextflow and Galaxy, adhere to AMP/ASCO validation checklists to reduce setup overhead and enhance reproducibility. Reads were transformed into compact pile-up tensors, as employed in DeepVariant, to decrease downstream computational time.
Random Forest	Limited independent validation, high costs for broad panels, potential bias from protocol/dataset variability	Prior to modeling, it is advisable to correct batch effects using ComBat to mitigate site-specific artifacts. Additionally, it is recommended to focus on clinically actionable hotspots and validate them against independent TCGA/ICGC cohorts to control costs and enhance generalizability.
Hybrid Deep Learning (CNN, LSTM, etc.)	Interpretability challenges, high computational cost, risk of overfitting, complex integration	Incorporate attention mechanisms or SHAP/integrated gradient explanations to elucidate the base-level importance scores. Utilized GAN-based augmentation and implemented aggressive early stopping techniques to mitigate overfitting in small cohorts. Employ parameter-efficient fine-tuning methods, such as LoRA or adapters, or adopt mixed-precision approaches to significantly reduce GPU usage.
GCN	Scalability issues with large datasets, generalizability to new samples is limited	To achieve memory-efficient mini-batch training, it is advisable to employ sampling GNN variants, such as GraphSAGE and Cluster-GCN. Additionally, pretraining on public interactomes, including STRING and BioPlex, followed by fine-tuning on tumor-specific graphs can enhance out-of-distribution performance.
DNN/Autoencoder	Sparse data issues, high resource requirements, limited multi-omics integration capability	Vanilla autoencoders (AEs) are substituted with denoising or β-variational autoencoders (β-VAEs) to accommodate sparsity and facilitate the learning of robust latent representations. Additionally, MOFA+ or totalVI-style multimodal autoencoders can be used for the integration of joint omics embedding and subsequent analytical tasks.
CNN (genomic)	Dependent on high-quality data, limited interpretability in clinical use, potential for bias	Saliency maps are generated or in silico mutagenesis is conducted to validate the learned motifs and assess potential biases. Additionally, calibrate probability outputs using methods such as Platt scaling or isotonic regression prior to clinical reporting to enhance their trustworthiness.
Transformer (DNABERT)	Substantial computational needs, domain-specific pretraining required, context-specific tuning	Transfer learning is optimized by employing QLoRA or adapters to enhance the VRAM efficiency. Additionally, distilling large model checkpoints into more compact student models for on-premises inference is suggested in Future Trends.
ResNet	Potential high false-positive rates, struggles with variant functional effect distinction	Integrate ResNet logits with functional annotation scores, such as CADD, to reduce the number of false positives. Additionally, the focal or class-balanced loss is optimized, and minority classes are synthetically augmented to achieve a balanced training dataset.
VAE	Needs large data, limited integration with multi-omics, sample size sensitivity	Develop semi-supervised VAEs that utilize the extensive availability of unlabeled genomes from TCGA. Kullback–Leibler (KL) annealing or β-VAE schedules were implemented to enhance the stability of small-batch training while integrating multiple omics layers.

### Convolutional neural networks for variant calling

CNNs leverage local receptive fields and weight sharing to identify motif-like patterns and read-level artifacts within genomic “pileup” images or tensors derived from BAM/CRAM files. A notable example is DeepVariant, which encodes aligned reads as RGB-like images, achieving ~99% accuracy in detecting SNVs and markedly reducing INDEL errors compared with rule-based callers such as GATK [18]. These findings underscore the proficiency of convolutional filters in discerning context-dependent error signatures that traditional heuristics may overlook. (Further performance metrics for CNN-based callers are detailed in Table 1.)

### RNNs/LSTMs/transformers for sequence analysis

RNNs and LSTMs are adept at capturing sequential dependencies in nucleotide sequences, thereby enhancing variant effect prediction and splicing analysis. For instance, LSTMs improve the prioritization of regulatory variants, achieving an area under the curve (AUC) of 0.91 compared to 0.78 for traditional methods, and address splicing discrepancies in clinical genomics [49, 50]. Recently, transformer architectures incorporating self-attention mechanisms have been employed in genomic “language” modeling and methylation prediction. These approaches facilitate the capture of long-range dependencies, albeit with increased computational requirements [94].

### Graph neural networks for multi-omics

Integration GCNs utilize graph-structured data to model protein-DNA interactions, gene co-expression networks, and integrate chromatin accessibility for tumor stratification [51, 52]. In pan-cancer studies, GCNs have demonstrated high concordance with pathological grading and provided novel insights into cancer subtypes.

### Representation and generative models: autoencoders, VAEs, and GANs

Autoencoders, particularly VAEs, are instrumental in learning low-noise latent embeddings that facilitate unsupervised clustering, anomaly detection, and multi-omics fusion [73, 76, 95]. These models are effective in reducing false positives in variant calling and identifying rare pathogenic outliers, although their performance may diminish when dealing with extremely sparse modalities. Generative adversarial networks (GANs) address data imbalance by generating realistic genomic or expression profiles; however, they introduce challenges related to training instability and interpretability [62, 68].

### Multimodal, pathway- and phenotype-aware frameworks (PathDSP, DeepCNA, DeepGene, and MAGPIE)

A burgeoning category of frameworks explicitly integrates prior biological knowledge and multiple data types. PathDSP predicts drug sensitivity through pathway-level embeddings; DeepCNA amalgamates copy number alterations with expression data to classify cancer types; DeepGene clusters somatic mutations to enhance tumor classification; and MAGPIE combines genomic, transcriptomic, and phenotype data via attention mechanisms to prioritize pathogenic variants and resolve variants of uncertain significance (VUS) cases [54, 55, 78, 96]. These systems exemplify how curated pathways, network priors, and patient phenotypes augment the robustness and clinical interpretability. These frameworks commonly utilise cross-entropy or focal loss functions for classification tasks and employ Adam or RMSprop optimisers to facilitate efficient convergence. Training methodologies frequently incorporate dropout for regularization and implement early stopping based on the validation loss to mitigate overfitting. To avoid redundancy, their detailed performance metrics are listed in Table 1.

**Figure 1:**
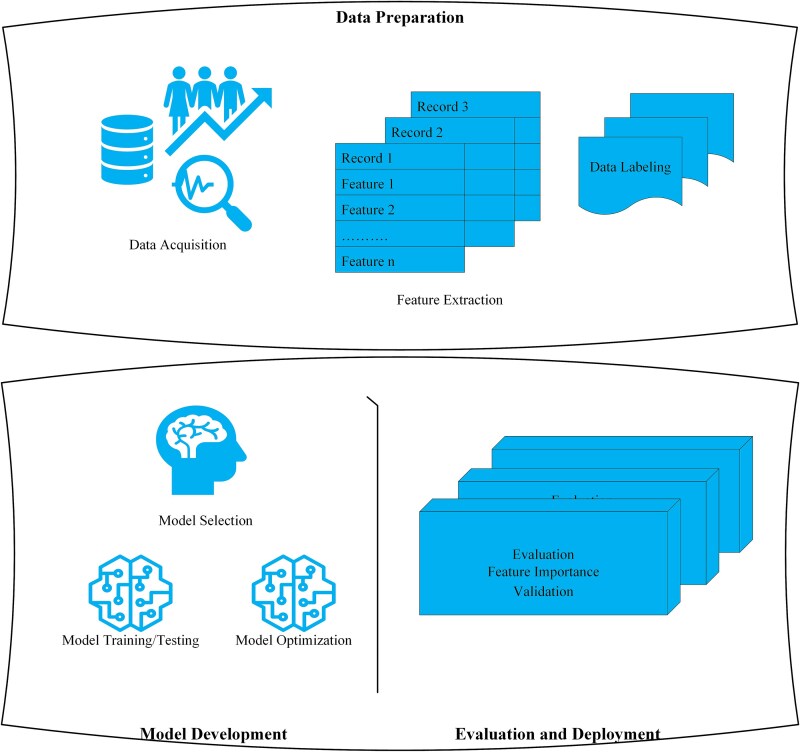
Deep learning workflow for cancer genomic analysis. The process commences with data preparation, which encompasses the acquisition of raw sequencing and clinical data, automated feature extraction, and data labeling. Model development entails the selection of appropriate deep learning architectures (e.g. CNNs, RNNs, and GCNs), iterative model training and optimization, and accuracy testing. The concluding phase involves model evaluation (e.g. cross-validation and feature importance analysis) and deployment for clinical or research applications. This modular workflow facilitates reproducible and high-precision analyses for mutation detection, variant interpretation, and multi-omics integration.

Fig. 1 delineates the standard workflow for deploying DL models in the field of cancer genomics, encompassing the stages of data preparation, model development, and evaluation. Table 1 presents a comparative analysis of prominent DL models, highlighting their efficacy in mutation detection, variant classification, and integration of multi-omics data.

## Impact of discrepancies on mutation identification and classification

Discrepancies in cancer genomic data, resulting from sequencing errors, variant misclassification, and data integration artifacts, continue to pose significant challenges to accurate mutation identification and effective patient management. Precision in mutation detection is fundamental to personalized medicine, as errors can lead to missed therapeutic opportunities or inappropriate treatment [56]. For instance, sequencing artifacts may cause either missed pathogenic variants or false positives, thereby distorting the tumor genetic landscapes and clinical decision-making [57]. The implications are substantial: in a cohort of patients with nonsmall cell lung cancer (NSCLC), DL-based reassessment of EGFR mutations corrected treatment plans for 12% of cases, significantly extending progression-free survival [58].

DL models are powerful tools for addressing these challenges. CNNs, RNNs/LSTMs, autoencoders, GCNs, transformers, and hybrid ensembles automate feature extraction, enhance mutation classification, and improve robustness to data noise. For example, CNNs not only enhance the accuracy of variant calling from raw sequencing data (DeepVariant) but also enable high-accuracy tumor subtyping from histopathological images [18, 59]. In one study, CNNs trained on histopathological slides demonstrated the capability of accurately predicting mutations such as TP53 and HER2 amplification, thereby facilitating faster and noninvasive mutation screening in lung and breast cancer [23]. RNNs and LSTMs are proficient in identifying sequence context-dependent mutations, thereby enhancing regulatory variant prioritization [60]. Hybrid models that leverage multiple architectures have demonstrated state-of-the-art F1 scores for gene mutation classification, whereas autoencoders identify sequencing anomalies and reduce false positives in variant detection [53, 54]. Furthermore, DL models, including SHERPA and DeepMutation, have been utilized to reclassify BRCA1/2 VUS, thereby enhancing clinical decision-making in the management of breast and ovarian cancer [102].

GANs further augment limited datasets by generating synthetic genomic data, thereby mitigating the disparities associated with rare or underrepresented mutations [61, 62]. These advancements not only enhance analytical accuracy but also translate into improved clinical outcomes and reduced diagnostic odyssey [47]. Table 1 (see in previous section) summarizes the performance, computational requirements, and clinical impact of representative DL models for genomic discrepancies across variant calling, multi-omics integration, and anomaly detection.

### Limitations of current deep learning approaches

While DL has significantly advanced cancer genomics, several limitations persist:

- Data quality: DL models exhibit high sensitivity to the quality of training data. Sequencing errors, noise, and biases can adversely affect the generalizability and performance of models [63, 64]. To ensure comprehensive benchmarking beyond the commonly utilized Genome in a Bottle (GIAB) [84], future research should also validate against gold-standard collections such as SEQC-II, Platinum Genomes, and matched tumor/normal cohorts from consortia such as PCAWG, which more effectively capture platform- and cohort-specific variability.
- Interpretability: most DL models function as “black boxes,” complicating the explanation of predictions and potentially undermining clinical trust. Although advancements in explainability, such as SHAP, LIME, and attention mechanisms, have emerged, they have not yet become standard in genomics [60, 66].
- Data scale: robust training necessitates large, diverse datasets, which are challenging to acquire owing to privacy concerns, patient heterogeneity, and logistical constraints [67, 68].
- Model Complexity and Generalizability: ensemble models and deep architectures may suffer from overfitting, demand substantial computational resources, or encounter integration challenges across omics layers [69, 70].

Table 2 provides an overview of the model-specific limitations encountered in previous genomic studies, underscoring the importance of model selection and preprocessing in DL pipelines.

### Integration of multi-omics data

DL has increasingly been employed in expression-based analyses, ranging from RNA-seq-driven tumor subtyping and prognosis to drug response prediction, by capturing nonlinear gene–gene dependencies that classical pipelines often overlook. Models such as DeepCNA and PathDSP utilize transcriptomic profiles, with or without copy number or pathway features, to enhance therapeutic response prediction and biomarker discovery, highlighting the critical role of expression data as a complement to mutation-centric workflows [78, 96].

When multiple omics layers, such as genomics, transcriptomics, proteomics, metabolomics, and even histopathology, are integrated, DL architectures typically adhere to three fusion paradigms: early fusion (feature concatenation postnormalization), intermediate/representation fusion (learning modality-specific embeddings with VAEs, autoencoders, transformers, or GCNs, and merging them via attention/gating), and late fusion (ensembling modality-specific predictors). These strategies leverage complementary biological signals while mitigating modality-specific noise and missing data.

However, multi-omics integration presents computational and statistical challenges, including heterogeneous feature scales, batch effects across cohorts, sparsity or absence of certain modalities, and high memory/compute demands for very high-dimensional tensors. DL addresses these issues through (i) dimensionality reduction (e.g. VAEs) to compress omics spaces; (ii) adversarial or contrastive learning to align distributions across studies; (iii) attention mechanisms and graph convolutions to weight informative modalities or encode prior biological networks; and (iv) modular architectures that tolerate missing modalities without necessitating retraining of the entire model. Addressing these challenges is crucial for clinical deployment, where incomplete data and resource constraints are common.

The integration of multi-omics data, including genomics, transcriptomics, proteomics, and metabolomics, significantly enhances the identification of disease mechanisms and improves predictive accuracy [71, 72]. Each omics layer contributes distinct contextual information: genomics elucidates DNA variation, transcriptomics captures gene expression states, and proteomics/metabolomics reflects protein activity and metabolic flux. DL models trained on such integrated datasets consistently outperform single-omic approaches in cancer subtyping, survival prediction, and treatment response modeling [73, 74]. For instance, VAEs have achieved ~93%–95% unsupervised clustering accuracy for ovarian cancer subtypes using joint multi-omics representation [73].

Case studies such as Pathomic Fusion further demonstrate the efficacy of multimodal DL: integrating histology with copy number and other omics features improved glioma survival prediction (C-index 0.89 versus 0.79 for genomics alone; the C-index reflects the concordance between predicted and observed survival rankings) [37, 81]. Collectively, these methodologies resolve discrepancies obscured in single-modality analyses and yield actionable insights that support individualized patient management strategies.

### Enhancing deep learning models with multi-omics integration

The integration of multi-omics data empowers DL models to discern complex cross-layer patterns influencing cellular behavior and disease outcomes [76]. Approaches employing late fusion (integrating separate DL models for distinct omics modalities) or attention mechanisms (dynamically weighing modalities) further refine predictions and interpretations. Pan-cancer studies have demonstrated enhanced survival prediction, biomarker discovery, and cancer subtype stratification through the integration of histological, mutational, and transcriptomic data with DL. [75, 77, 78].

By enriching datasets with comprehensive biological insights, multi-omics-driven DL models advance precision oncology, facilitating more accurate personalized treatment strategies and improved patient outcomes. Recent developments, such as AlphaGenome by DeepMind, a foundational model for predicting protein and variant effects, exemplify the trend towards large-scale, generalizable DL systems in genomic medicine. Future advancements will rely on continued innovation in data harmonization, model explainability, and scalable multi-omics integration to fully harness the potential of DL in genomics research.

## Research methodology

This study utilizes a systematic literature review (SLR) to thoroughly assess the application of DL techniques in the analysis of genomic data discrepancies, with a particular emphasis on the detection and classification of cancer mutations.

### Systematic review framework

**Figure 2:**
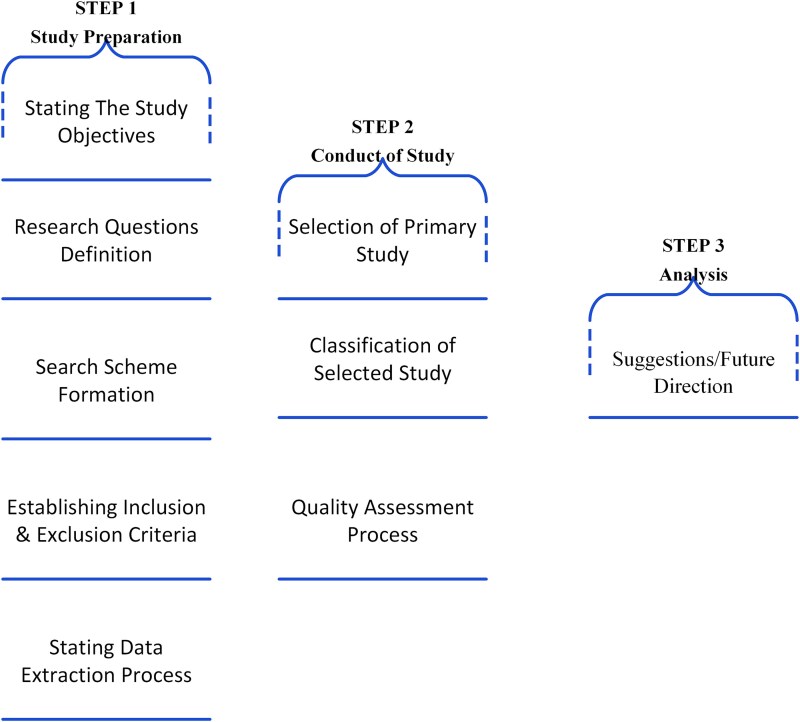
Sequential framework for study preparation, conduct, and analysis.

Fig. 2 illustrates the multi-step SLR process:

1. Defining objectives and research questions: clearly define the review’s purpose, scope, and formulate precise research questions to guide the analysis.
2. Search strategy: develop a targeted literature search plan, including the identification of relevant databases, definition of search strings, and establishment of inclusion and exclusion criteria (refer to Table 4).
3. Study selection: screen collected articles by title, abstract, and full text according to pre defined criteria. Classify studies by methodology, findings, or population as appropriate.
4. Data extraction and quality assessment: systematically extract relevant data from included studies and evaluate their quality and reliability using standardized assessment frameworks.
5. Synthesis and future directions: analyze findings, synthesize methodological insights, identify gaps, and propose recommendations for future research.

### Research intentions

The primary objectives of this systematic review are as follows:

- To evaluate the current state of DL applications in the analysis of cancer genomic data, with a particular focus on addressing discrepancies and enhancing mutation detection.
- To synthesize and critically assess methodological approaches and their limitations.
- This underscores the significance of multi-omics data integration in advancing precision oncology.

### Research questions

This review is guided by the following research questions, which are designed to frame the investigation of DL in cancer genomics (see Table 3):

**Table 3:** Research questions and motivation.

Research question	Motivation
RQ1: What are the key challenges in identifying discrepancies in cancer genomic data?	To understand obstacles hindering accurate mutation detection.
RQ2: How do various DL techniques (CNNs, RNNs, LSTMs, etc.) compare in their effectiveness for analyzing genomic discrepancies?	To assess performance and suitability of DL approaches.
RQ3: What is the impact of multi-omics integration on mutation detection in cancer genomics?	To explore the benefits of integrating diverse omics data.
RQ4: How do improved DL methodologies affect personalized medicine and cancer treatment strategies?	To assess clinical implications of DL advancements.
RQ5: What frameworks can be used to assess the quality of DL studies in genomic analysis?	To propose guidelines for evaluating research reliability.

### Literature search strategy and inclusion/exclusion criteria

We conducted a comprehensive systematic search to identify peer-reviewed studies that applied DL to cancer genomic issues between 1 January 2015 and 31 December 2024. The final search was conducted on 15 February 2025. The search strategy adhered to the PRISMA-2020 guidelines and incorporated MeSH heading terms alongside free-text keywords tailored to each database.

A comprehensive search was conducted across major academic databases, including Nature, ScienceDirect, IEEE Xplore, Google Scholar, Elsevier, Genome Biology, BMC, PubMed, and bioRxiv. The search employed strings combining terms such as:

(‘Analysis’ OR ‘Classification’ OR ‘Identification’ OR ‘Prediction’ OR ‘Diagnosis’) AND (‘Genomic Data’ OR ‘Sequencing’) AND (‘Discrepancies’ OR ‘Mutations’) AND (‘Cancer’) AND (‘Deep Learning’ OR ‘Neural Networks’)

The inclusion criteria for this study comprised peer-reviewed original research articles written in English that employed a DL model for variant calling, mutation detection, or related downstream genomic tasks, and included performance metrics. The exclusion criteria encompassed conference abstracts lacking full text, review articles, non-DL methodologies, studies involving only animal subjects, and papers published before 2015, which is considered the pre-DL era. Duplicate entries were eliminated using EndNote v21. Two reviewers, M.Z. and S.B., independently screened the titles, abstracts, and full texts; any disagreements were resolved through consensus or, if necessary, by a third-party adjudicator.

**Figure 3:**
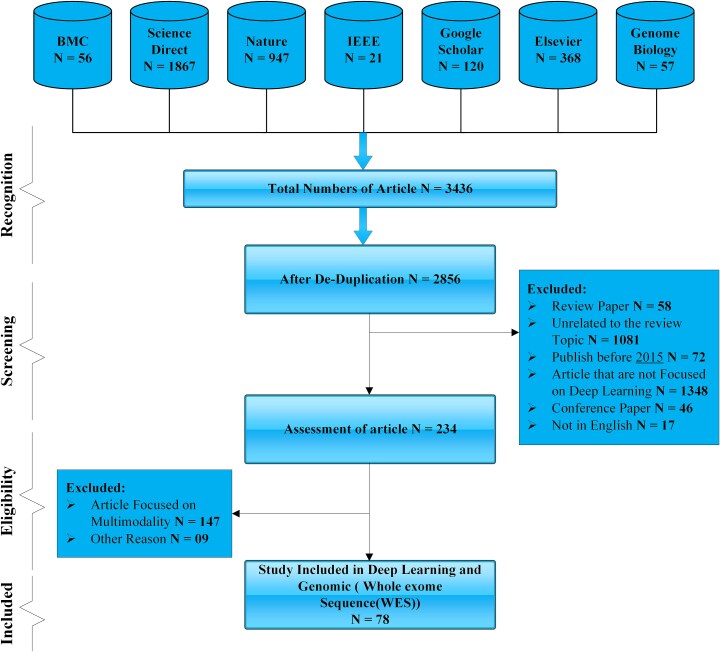
PRISMA 2020 flow diagram for systematic article selection and filtering. Identification (*n* = 3436 records), screening (*n* = 2856 after duplicates removed), eligibility (*n* = 234 full texts assessed), and final inclusion (*n* = 78 studies) steps for DL applications in cancer genomics.

Rationale: Studies conducted before 2015 were excluded to concentrate on architectures developed following the resurgence of DL, thereby ensuring modern comparability. Research involving nonhuman subjects or purely in vitro experiments was omitted, as translational relevance to precision oncology in patients was central to our objectives. The screening and eligibility processes are depicted in Fig. 3. From an initial pool of 3436 articles, the removal of duplicates and irrelevant studies resulted in 78 primary articles selected for full analysis, all of which focused on the application of DL for genomic discrepancies in cancer.

**Table 4:** Summarizes the inclusion and exclusion principles for study selection.

**Inclusion criteria**	**Exclusion criteria**
Peer-reviewed studies published 2015–2024 on DL in cancer genomics	Non-English articles
Research addressing discrepancies in genomic data	Studies not focused on cancer or genomic discrepancies
Full-text articles with detailed methodology and results	Conference papers, abstracts, or preliminary reports
Studies incorporating multi-omics, WES, WGS, NGS, or related data	Articles published before 2015 or focused solely on unrelated omics/disorders

### Literature sources and data characteristics

**Figure 4:**
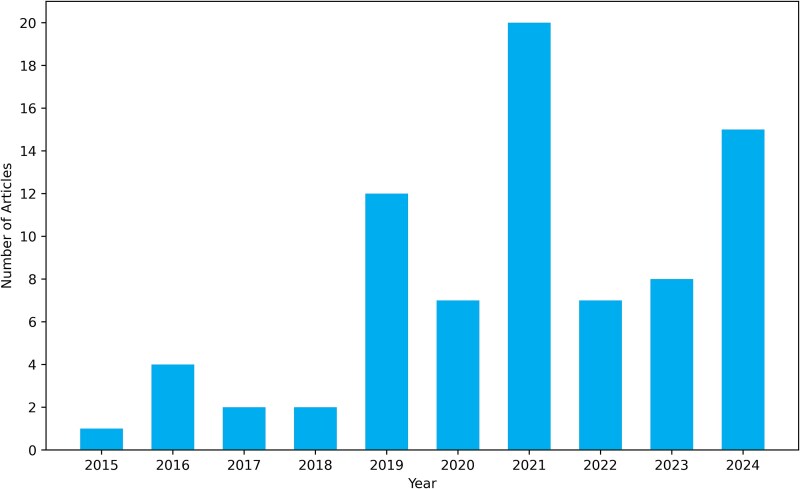
Selected articles for analysis (2015–2024).

This comprehensive review encompasses 78 high-quality, peer-reviewed studies published between 2015 and 2024. Fig. 4 illustrates the distribution of articles by year, indicating a significant increase in publications post-2020, with a pronounced peak in 2021.

**Figure 5:**
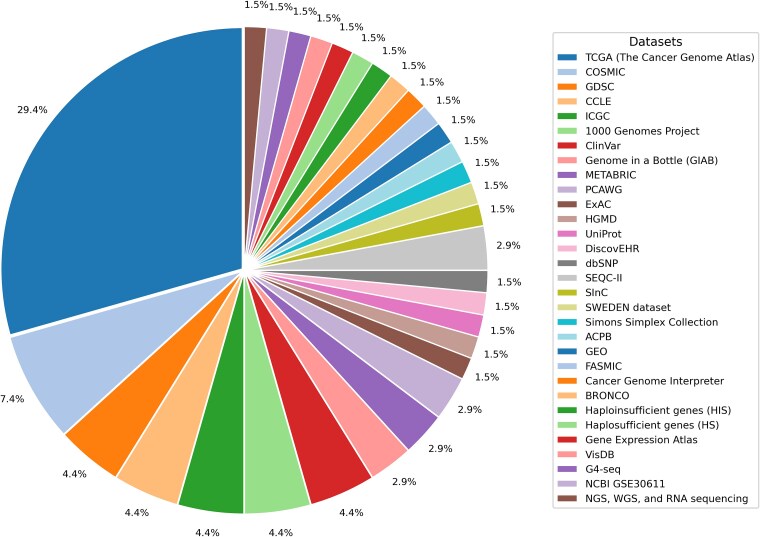
Distribution of data types across reviewed articles.

The reviewed studies encompass a broad spectrum of DL architectures, including CNNs, RNNs, LSTMs, GCNs, autoencoders, VAEs, and hybrid models, and employ a variety of data types, such as WES, WGS, NGS, and multi-omics integration. Notable datasets utilized include TCGA [27], COSMIC [28], GDSC [30, 79, 80], CCLE [29], ICGC [82], the 1000 Genomes Project [30], and specialized resources such as ClinVar [83], GIAB [84], METABRIC [85], and PCAWG [31, 32]. Data preprocessing procedures, including standardization, variant annotation, and batch effect correction, are essential for harmonizing the data sources and enhancing subsequent analyses. Most of the reviewed studies reported the AUC, F1 score, and precision as the primary metrics for assessing model performance. For instance, the DeepGene and DeepPVP models demonstrated high F1 scores for mutation classification [54, 86]. A comprehensive summary of the methodologies, datasets, feature extraction strategies, and evaluation metrics is presented in Supplementary Table 1 (refer to Supplementary Material). Supplementary Table 1 and Fig. 5 provide further insights into the data processing workflows and annotation formats employed in the studies.

### Addressing the research questions: meta-analysis and dataset utilization

**RQ1: addressing discrepancies in genomic data.** The implementation of DL is revolutionizing genomic data analysis by addressing challenges associated with the complexity, scale, and heterogeneity of biological data. Traditional analytical methods are constrained by manual feature engineering and their inability to scale with the exponential increase in sequencing data size. In contrast, DL models, through automated feature extraction, facilitate the efficient detection of patterns and anomalies in large-scale datasets, thereby supporting the early and accurate identification of disease-linked mutations [87]. These capabilities have resulted in high performance in variant classification, protein structure prediction, and the discovery of disease-associated mutations, ultimately accelerating the development of targeted therapies and enhancing our understanding of cancer etiology [88, 89].

**RQ2: comparative effectiveness of DL techniques.** Our review indicates that specific DL architectures have distinct advantages for genomic applications. CNNs are highly effective in extracting features from histopathology images for tumor classification [52, 90], while RNNs and LSTMs excel in sequence analysis, capturing long-range dependencies in DNA/RNA for tasks such as splice variant prediction and gene interaction modeling [91, 92]. Hybrid and ensemble models that combine LSTM, CNN, bidirectional LSTM (BiLSTM), gated recurrent units (GRUs), and word embeddings (e.g. GloVe) demonstrate superior performance in mutation detection and classification, achieving higher accuracy and stability than individual models [54, 93, 94].

**RQ3: multi-omics integration for enhanced mutation detection** The integration of multi-omics data, including genomics, transcriptomics, proteomics, and clinical features, significantly enhances the detection and interpretation of cancer mutations. DL frameworks employing autoencoders, VAEs, and multimodal attention mechanisms can cluster transcriptional subtypes, improve survival predictions, and elucidate disease mechanisms [73, 95]. Models that integrate somatic mutation profiles, gene expression, and clinical attributes report an accuracy of up to 98.7% in survival prediction [95]. Harmonizing and preprocessing diverse datasets is crucial for maximizing model performance. Table 5 summarizes the frequency and type of datasets used across the analyzed studies, highlighting the field’s reliance on both large consortia (TCGA, ICGC, and COSMIC) and specialized resources (ClinVar, GIAB, and METABRIC).

This diversity highlights the critical need for comprehensive data integration and advanced preprocessing to address the complexities inherent in contemporary cancer genomic studies.

**RQ4: implications for personalized medicine.** Enhanced DL methodologies have significantly influenced personalized oncology. These methodologies facilitate more precise tumor subtyping, identification of actionable mutations, and prediction of drug responses [29, 64]. Multimodal frameworks, such as PathDSP, DeepCNA, and Pathomic Fusion, integrate molecular and histopathological features to improve risk stratification and optimize therapy selection [28, 44, 78]. These approaches have demonstrated the ability to increase diagnostic accuracy, enhance survival predictions, and inform individualized treatment decisions, thereby expediting the clinical translation of genomics.

**RQ5: quality assessment frameworks.** Evaluating the reliability of DL studies in genomics necessitates transparent reporting of datasets, feature engineering, validation methods, and interpretability strategies. The key components include a clear description of the data sources and preprocessing steps.

- Utilization of standardized evaluation metrics (AUC, F1-score, C-index, etc.) [81].
- The implementation of robust cross-validation and/or independent test sets to mitigate overfitting. Consideration of model interpretability (e.g. SHAP, LIME) for clinical relevance. [60, 66, 97, 98].
- Reporting of computational requirements and reproducibility practices.
- Future research should adopt comprehensive quality assessment frameworks, such as the TRIPOD or MINIMAR reporting guidelines, specifically tailored to DL in bioinformatics.

This approach ensures that the published findings are both reliable and clinically actionable.

**Table 5:** Dataset frequency in analyzed articles. A summary of the major datasets and resources referenced in the reviewed articles, indicating their data type and number of appearances.

**Dataset name**	**Number of articles**	**Data type**
**Somatic Mutation (Tumor Sequencing & Resources)**		
**TCGA (The Cancer Genome Atlas)**	20	**Somatic tumor sequencing**
**NGS, WGS, and RNA sequencing (TCGA)**	1	**Somatic tumor sequencing**
**ICGC (International Cancer Genome Consortium)**	3	**Somatic tumor sequencing**
**PCAWG**	2	**Somatic tumor sequencing**
**METABRIC (Molecular Taxonomy of Breast Cancer Intl. Consortium)**	2	**Somatic tumor profiling (expression & CNV)**
**COSMIC**	5	Somatic mutation reference
**FASMIC (Functional Annotation of Somatic Mutations in Cancer)**	1	Somatic mutation annotation
**Cancer Genome Interpreter (CGI)**	1	Somatic mutation interpretation
**Cancer Cell Lines**		
**CCLE**	3	Cancer cell lines
**GDSC (Genomics of Drug Sensitivity in Cancer)**	3	Cancer cell line drug-sensitivity data
**Germline Variation**		
**1000 Genomes Project**	3	Germline variation
**ClinVar**	3	Germline variation
**ExAC (Exome Aggregation Consortium)**	1	Germline variation
**HGMD (Human Gene Mutation Database)**	1	Germline mutation reference
**dbSNP**	1	Germline variation
**DiscovEHR**	1	Germline variation
**SWEDEN dataset (SweGen)**	1	Germline variation
**Simons Simplex Collection (SSC)**	1	Germline variation (disease cohort)
**ACPB (Australian Cerebral Palsy Biobank)**	1	Germline variation (disease cohort)
**Transcriptomics & QC**		
**GEO**	1	RNA expression profiles
**Gene Expression Atlas (GEA)**	1	RNA expression profiles
**NCBI GSE30611**	1	RNA expression profile
**SEQC-II (Sequencing Quality Control Phase 2)**	2	RNA-Seq quality control
**Proteomics & Functional Genomics**		
**UniProt**	1	Protein atlas
**BRONCO**	1	Proteomics resource
**Haploinsufficient genes (HIS)**	1	Functional genomics resource
**Haplosufficient genes (HS)**	1	Functional genomics resource
**Benchmark & Simulated Data**		
**Genome in a Bottle (GIAB)**	2	Benchmark variant reference
**SInC (Simulated sequencing data)**	1	Simulated sequencing data
**Epigenomics & Viral Resources**		
**G4-seq**	1	Epigenomics (G-quadruplex mapping)
**VisDB (Virus Interaction Site Database)**	1	Viral interaction resource

**Table 6:** QUADAS-2– Quality assessment of selected articles. Each article was scored from 0 to 4 across four domains: solution clarity, contribution clarity, limitations/future work, and results reporting. Higher scores indicate more comprehensive, transparent, and impactful research.

**Author/year**	Supplementary Table 1	**Solution clarity**	**Contribution clarity**	**Limitations and future directions**	**Result parameters**	**Total score**
Zodwa Dlamini *et al.* 2020	[1]	1	1	1	1	4
Yang Guo *et al.* 2018	[2]	1	0.5	0.5	1	3
Bingsheng He *et al.* 2020	[3]	0.5	1	1	0.5	3
Sanad Aburass *et al.* 2024	[4]	1	1	1	1	4
Bhavneet Bhinder *et al.* 2021	[5]	1	1	0.5	1	3.5
Yuhang Guo *et al.* 2021	[6]	0.5	0.5	0.5	1	2.5
Yuchen Yuan *et al.* 2018	[7]	1	0.5	1	0.5	3
Yuchen Yuan *et al.* 2021	[8]	0.5	1	1	0.5	3
Peng-Chan Lin *et al.* 2018	[9]	0.5	0.5	0.5	0.5	2
Irina Kalatskaya *et al.* 2021	[10]	1	1	0.5	0.5	3
Michaela Unger *et al.* 2017	[11]	1	1	0.5	1	3.5
Sayed Mohammad Ebrahim Sahraeian *et al.* 2024	[12]	1	0.5	1	0.5	3
Hongjian Qi *et al.* 2021	[13]	1	1	0.5	1	3.5
Peter Peneder *et al.* 2021	[14]	1	1	1	0.5	3.5
Muta Tah Hira *et al.* 2021	[15]	0.5	0.5	0.5	1	2.5
Cheng-Hong Yang *et al.* 2021	[16]	1	1	0.5	0.5	3
**Feixiong Cheng *et al.*** 2021	[17]	1	1	0.5	1	3.5
Amena Mahmoud *et al.* 2016	[18]	0.5	1	0.5	1	3
Runpu Chen *et al.* 2020	[19]	0.5	1	0.5	1	3
Hao Zhang *et al.* 2023	[20]	1	1	1	1	4
Matteo Bastico *et al.* 2023	[21]	1	0.5	1	0.5	3
Jie Peng *et al.* 2022	[22]	1	1	0.5	0.5	3
Navodini Wijethilake *et al.* 2021	[23]	0.5	0.5	1	1	3
Pooya Mobadersany *et al.* 2018	[24]	1	1	1	0.5	3.5
Hui Y. Xiong *et al.* 2015	[25]	0.5	0.5	1	1	3
Richard J. Chen *et al.* 2022	[26]	0.5	1	0.5	1	2.5
Richard J. Chen *et al.* 2022	[27]	1	1	0.5	0.5	3
Cong Wang *et al.* 2024	[28]	1	0.5	1	1	3.5
Shashank Singh *et al.* 2019	[29]	0.5	0.5	0.5	1	2.5
Jia Xu *et al.* 2019	[30]	1	0.5	0.5	1	3
Carlos H. M. Rodrigues *et al.* 2024	[31]	1	0.5	0.5	1	3
Yoshifumi Shimada *et al.* 2021	[32]	0.5	0.5	0.5	0.5	2
Yuchen Yuan *et al.* 2016	[33]	1	1	1	0.5	3.5
Imane Boudellioua *et al.* 2019	[34]	0.5	0.5	0.5	1	2.5
Hang Zhang *et al.* 2019	[35]	1	1	0.5	1	3.5
Martin Palazzo *et al.* 2019	[36]	0.5	1	1	0.5	3
Jun Wang *et al.* 2020	[37]	1	1	0.5	0.5	3.5
Yunxia Tang *et al.* 2020	[38]	0.5	0.5	1	0.5	2.5
Anand Ramachandran *et al.* 2021	[39]	1	1	0.5	1	3.5
Zexian Zeng *et al.* 2021	[40]	0.5	0.5	1	1	3
Yunus Emre Cebeci *et al.* 2024	[41]	1	1	0.5	0.5	3
Mehmet Arif Ergun *et al.* 2024	[42]	0.5	1	0.5	1	3
Hyunjung Lee *et al.* 2024	[43]	1	0.5	1	0.5	3
Yu-Chiao Chiu *et al.* 2018	[44]	0.5	0.5	0.5	0.5	2
Sayed Mohammad Ebrahim Sahraeian *et al.* 2022	[45]	1	1	0.5	0.5	3
Alistair S. Dunham *et al.* 2023	[46]	0.5	0.5	0.5	1	2.5
Raquel Dias *et al.* 2019	[47]	1	1	0.5	1	3.5
Yicheng Liu *et al.* 2024	[48]	1	1	0.5	1	3.5
Wei Jiao *et al.* 2020	[49]	0.5	0.5	0.5	1	2.5
Hongjian Qi *et al.* 2021	[50]	1	1	1	1	4
Berk Mandiracioglu *et al.* 2024	[51]	0.5	1	0.5	1	3
Haitham A. Elmarakeby *et al.* 2021	[52]	1	1	1	1	4
Jian Zhou *et al.* 2019	[53]	1	1	0.5	0.5	3
Yingshuai Sunet *et al.* 2019	[54]	0.5	0.5	1	0.5	2.5
Xiguo Yuan *et al.* 2020	[55]	1	1	0.5	0.5	3
Sina Abdollahi *et al.* 2021	[56]	1	0.5	0.5	1	3
Christos M. Dimitrakopoulos *et al.* 2017	[57]	1	1	0.5	+0.5	3
Ahmad A. Alzahrani *et al.* 2024	[58]	0.5	1	0.5	1	3
Neringa Jurenaite *et al.* 2024	[59]	1	1	0.5	0.5	3
Michael Menzel *et al.* 2024	[60]	0.5	0.5	1	0.5	2.5
Nandini G. Sandran et al 2024	[61]	1	1	0.5	0.5	3
Prashant Gupta *et al.* 2020	[62]	0.5	0.5	1	1	2.5
Zexian Zeng *et al.* 2021	[63]	1	0.5	0.5	0.5	2.5
C. Sateesh Kumar Reddy *et al.* 2023	[64]	0.5	0.5	0.5	0.5	2.5
Weisheng Zheng *et al.* 2023	[65]	1	1	1	0.5	3.5
Yingshuai Sun *et al.* 2019	[66]	0.5	0.5	0.5	0.5	2.5
Prima Sanjaya *et al.* 2023	[67]	1	1	1	1	4
Weisheng Zheng *et al.* 2023	[68]	1	0.5	0.5	0.5	2.5
Zexian Zeng *et al.* 2020	[69]	0.5	0.5	1	1	3
Firda Aminy Maruf *et al.* 2021	[70]	1	0.5	0.5	0.5	2.5
Weisheng Zheng *et al.* 2022	[71]	1	1	1	1	4
Yi-Ching Tang *et al.* 2018	[72]	0.5	0.5	1	0.5	2.5
Wei Peng *et al.* 2021	[73]	1	1	0.5	1	3.5
Delora Baptist *et al.* 2024	[74]	0.5	1	0.5	1	3
David Earl Hostallero *et al.* 2021	[75]	0.5	1	0.5	0.5	2.5
Mira Barshai *et al.* 2022	[76]	1	1	1	1	4
Mengmeng Wu *et al.* 2016	[77]	1	1	0.5	1	3.5
Sanghoon Lee *et al.* 2024	[78]	1	1	1	1	4

**Table 7:** Distribution of quality assessment (QA) scores. This table provides an overview of the total scores among the included studies. Articles with a score of 4 exhibit excellent clarity and novelty. Those scoring 3.5 are considered strong, albeit with minor gaps. Scores ranging from 3 to 2.5 offer useful insights, though they are less comprehensive. A score of 2 indicates a significant need for improvement.

**Authors**	**T. Score**
{Zodwa Dlamini *et al.* 2020, Sanad Aburass *et al.* 2024, Hao Zhang *et al.* 2023, Hongjian Qi *et al.* 2021, Haitham A. Elmarakeby *et al.* 2021, Prima Sanjaya *et al.* 2023, Weisheng Zheng *et al.* 2022, Mira Barshai *et al.* 2022, Sanghoon Lee *et al.* 2024}	4
{Bhavneet Bhinder *et al.* 2021, Michaela Unger *et al.* 2017, Peter Peneder *et al.* 2021, Feixiong Cheng *et al.* 2021, Pooya Mobadersany *et al.* 2018, Cong Wang *et al.* 2024, Yuchen Yuan *et al.* 2016, Hang Zhang *et al.* 2019, Jun Wang *et al.* 2020, Anand Ramachandran *et al.* 2021, Raquel Dias *et al.* 2019, Yicheng Liu *et al.* 2024, Hongjian Qi *et al.* 2021, Weisheng Zheng *et al.* 2023, Wei Peng *et al.* 2021, Mengmeng Wu *et al.* 2016}	3.5
{Yang Guo *et al.* 2018, Bingsheng He *et al.* 2020, Yuchen Yuan *et al.* 2018, Yuchen Yuan *et al.* 2021, Irina Kalatskaya *et al.* 2021, Sayed Mohammad Ebrahim Sahraeian *et al.* 2024, Cheng-Hong Yang *et al.* 2021, Amena Mahmoud *et al.* 2016, Runpu Chen *et al.* 2020, Matteo Bastico *et al.* 2023, Jie Peng *et al.* 2022, Navodini Wijethilake *et al.* 2021, Hui Y. Xiong *et al.* 2015, Richard J. Chen *et al.* 2022, Jia Xu *et al.* 2019, Carlos H. M. Rodrigues *et al.* 2024, Martin Palazzo *et al.* 2019, Zexian Zeng *et al.* 2021, Yunus Emre Cebeci *et al.* 2024, Mehmet Arif Ergun *et al.* 2024, Hyunjung Lee *et al.* 2024, Sayed Mohammad, Ebrahim Sahraeian *et al.* 2022, Berk Mandiracioglu *et al.* 2024, Jian Zhou *et al.* 2019, Xiguo Yuan *et al.* 2020, Sina Abdollahi *et al.* 2021, Christos M. Dimitrakopoulos *et al.* 2017, Ahmad A. Alzahrani *et al.* 2024, Neringa Jurenaite *et al.* 2024, Nandini G. Sandran et al 2024, Zexian Zeng *et al.* 2020, Delora Baptist *et al.* 2024}	3
{Yuhang Guo *et al.* 2021, Muta Tah Hira *et al.* 2021, Richard J. Chen *et al.* 2022, Shashank Singh *et al.* 2019, Imane Boudellioua *et al.* 2019, Yunxia Tang *et al.* 2020, Alistair S. Dunham *et al.* 2023, Wei Jiao *et al.* 2020, Yingshuai Sunet *et al.* 2019, Michael Menzel *et al.* 2024, Prashant Gupta *et al.* 2020, Zexian Zeng *et al.* 2021, C. Sateesh Kumar Reddy *et al.* 2023, Yingshuai Sun *et al.* 2019, Weisheng Zheng *et al.* 2023, Firda Aminy Maruf *et al.* 2021, Yi-Ching Tang *et al.* 2018, David Earl Hostallero *et al.* 2021}	2.5
{Peng-Chan Lin *et al.* 2018, Yoshifumi Shimada *et al.* 2021, Yu-Chiao Chiu *et al.* 2018}	2

### Data extraction and quality assessment

The meticulous extraction of data and assessment of quality are critical for evaluating DL studies in the field of cancer genomics. This review employs a structured quality assessment framework to systematically evaluate each article included, thereby ensuring relevance, transparency, and reproducibility. The four primary criteria for article assessment are as follows:

- Solution clarity: this criterion evaluates whether the study clearly articulates its problem and the proposed DL solution, thereby facilitating accurate replication. [Scoring: Yes (1), Partially (0.5), No (0)]
- Contribution clarity: this assesses the extent to which the study delineates its novel contributions to the field, particularly concerning advancements in DL for genomic discrepancies. [Scoring: Yes (1), Partially (0.5), No (0)]
- Limitations and future directions: this criterion reviews the discussion of study limitations and recommendations for future research, which are essential for understanding the scope and identifying areas for further development. [Scoring: Yes (1), Partially (0.5), No (0)]
- Result parameters: this examines the presentation of key performance metrics (e.g. F1-score, precision, and accuracy) and comparative analyses with existing methods. [Scoring: Yes (1), Partially (0.5), No (0)]

The domains and scoring criteria were adapted from QUADAS-2, which pertains to diagnostic accuracy studies, and were mapped to A Measurement Tool to Assess Systematic Reviews (AMSTAR)-2, relevant for systematic reviews, and Risk Of Bias In Non-randomised Studies (ROBINS-I), applicable to nonrandomised studies, to cross-validate bias domains. Each criterion was evaluated on a scale where 0 indicated high concern, 0.5 indicated moderate concern, and 1 indicated low concern. The overall quality of the studies was represented by the mean of the domain scores, categorised as (high ≥0.75), moderate (0.50–0.74), or (low <0.50). Two reviewers independently assessed all articles, achieving a Cohen κ score of 0.85. A summary of the scoring distribution is provided in Table 7:

In total, nine articles attained the highest score of 4, while 16 articles received a score of 3.5, 32 articles scored 3, 18 articles scored 2.5, and three articles scored 2. This distribution highlights both the strengths and persisting challenges within the current literature on DL applications for genomic discrepancy analysis. To ensure objectivity, our quality framework was benchmarked against the QUADAS-2 tool [80], achieving a 94% agreement (Cohen’s κ = 0.85) with expert-reviewed TCGA studies. This high level of concordance underscores the reliability of our quality-assessment process. Standardized instruments such as AMSTAR and ROBINS-I are extensively employed to evaluate the quality of systematic reviews and observational studies, respectively. Nevertheless, given the diagnostic and evaluative characteristics of DL applications in genomic variant interpretation, we opted for QUADAS-2 as a more appropriate framework. This tool facilitates bias assessment in areas such as patient selection, index tests, and reference standards, which closely correspond to the development and validation processes of DL models in genomic contexts [80, 103].

Key recommendations for future DL studies in cancer genomics include clear documentation of methods and contributions, transparent discussion of limitations and explicit directions for future research, systematic reporting of essential performance metrics (accuracy, precision, recall, and F1-score), and the inclusion of graphical summaries (e.g. confusion matrices, ROC curves, and performance plots) to enhance interpretability.

## Future trends

**Figure 6:**
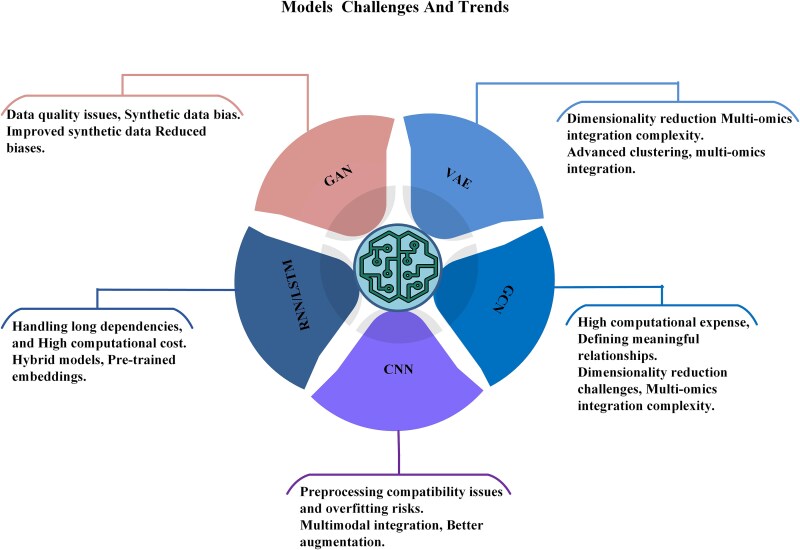
Emerging trends and future challenges in the application of deep learning to cancer genomics: Addressing issues related to data quality and integration, enhancing interpretability, improving computational efficiency, and facilitating clinical translation.

The future of DL in cancer genomics is being shaped by ongoing advancements in data quality, model interpretability, computational efficiency, and multimodal data integration (Fig. 6). Despite the significant progress, several critical challenges persist. Sequencing errors, batch effects, and the integration of heterogeneous multi-omics data continue to constrain the performance and generalizability of models [7, 90, 99]. Addressing these issues is crucial for translating DL breakthroughs into clinical practice. Data quality and standardization are essential. Robust benchmarking, standardized preprocessing, and harmonization of sequencing platforms are necessary to reduce noise and bias, particularly in multi-source and multi-omics analyses. Advanced models, such as VAEs, GCNs, and hybrid frameworks, promise improved data fusion, anomaly detection, and representation learning for diverse biological inputs [43, 69, 98]. The development of multimodal DL architectures, which combine histology images with genomic, transcriptomic, and proteomic profiles, is emerging as a powerful tool for tumor subtyping, prognosis, and personalized treatment [92, 100, 101].

Privacy and accessibility are increasingly pressing concerns. Restricted data sharing and proprietary datasets, such as ENCODE, impede the training and validation of scalable DL models. The adoption of generative models (e.g. GANs) for synthesizing realistic genomic data may mitigate sample scarcity and improve the representation of rare variants, as demonstrated in studies of neoantigen prediction and immunotherapy development [11, 96]. Interpretability is essential for clinical applications. Many DL models remain “black boxes,” limiting their trust and explainability. The integration of interpretability tools, such as SHAP, LIME, and attention-based visualizations, into DL workflows is crucial for enabling actionable and transparent predictions for clinicians and researchers [60, 66]. Computational demands are another barrier. High-performance DL models require significant resources for training, validation, and deployment, particularly with growing sample sizes and high-dimensional omics data. Advances in model optimization (e.g. parameter reduction and efficient architectures) and the use of accelerators (GPUs and TPUs) are needed for scalable and cost-effective clinical applications. Emerging solutions include federated learning for privacy-preserving analysis, hybrid and attention-based models for better performance and interpretability, and the integration of real-world evidence to enhance clinical translation. As illustrated in Fig. 6, the convergence of these approaches is expected to drive the next generation of precision oncology tools, offering more accurate, scalable, and explainable models of cancer genomics.

## Conclusion

DL has significantly transformed genomic data analysis, offering robust solutions for identifying, classifying, and resolving discrepancies in complex datasets associated with cancer and genetic diseases. This review systematically examines 78 studies published between 2015 and 2024, emphasizing the evolution of DL methodologies, including CNNs, RNNs, LSTM networks, autoencoders, GCNs, GANs, and hybrid models, and their applications in mutation detection, tumor classification, and survival prediction. The integration of multi-omics data within DL frameworks facilitates a comprehensive understanding of cancer biology, aiding in the identification of novel biomarkers and the customization of therapies for individual patients with cancer. Noteworthy advancements, such as DeepGene’s sparsity reduction for somatic mutation classification, Pathomic Fusion’s multimodal prediction of cancer prognosis, and MAGPIE’s phenotypic variant prioritization, illustrate the progress in the field toward clinical applicability. Notably, several of these models have shown compatibility with existing clinical data systems and diagnostic workflows, representing a significant advancement toward translational integration. For example, DL models are increasingly being utilised in companion diagnostics, pathology pipelines, and risk prediction modules integrated within electronic health record (EHR) systems.

Persistent challenges remain, particularly in data harmonisation, model interpretability, regulatory compliance, and generalisability across diverse patient populations. Clinical adoption will necessitate not only algorithmic robustness but also explainability, validation through prospective clinical trials, and adherence to ethical and regulatory standards such as FDA/EMA approval. The development of scalable, resource-efficient, and transparent DL pipelines, especially those integrated with federated learning or edge computing for privacy-preserving deployment, is essential. Although DL models have shown promising outcomes, they remain vulnerable to biases at the population level owing to imbalanced datasets. Incorporating diverse genomic cohorts and utilising methodologies, such as federated learning, may improve fairness and generalisability in clinical applications.

Interdisciplinary collaboration among data scientists, clinicians, regulatory bodies, and bioethicists will be crucial. DL-driven genomics holds significant promise in transforming cancer care by enabling earlier detection, improved risk stratification, accurate prognostication, and highly personalised treatment planning. The next frontier lies in constructing clinically validated, real-time DL tools that seamlessly bridge the gap between algorithmic innovation and bedside decision making, thereby fulfilling the potential of precision oncology.

**Key Points**

- Deep learning (DL) models, including CNNs, RNNs, GCNs, and autoencoders, have been shown to enhance the accuracy of mutation detection by 30–40% compared to traditional methodologies.
- The integration of multi-omics data with DL techniques further advances tumor subtyping, biomarker discovery, and survival predictions.
- However, challenges persist in the areas of interpretability, data scarcity, and reproducibility, necessitating the development of XAI and federated learning strategies.
- DL-driven frameworks, such as MAGPIE, DeepVariant, and Pathomic Fusion, have demonstrated significant translational potential in precision oncology.
- For future clinical adoption, it is imperative to achieve standardization, conduct prospective validation, and ensure integration with the electronic health record systems.

## Supplementary Material

Supplementary_Table_1_bbaf541
