# Supplementary material for: Deep learning approaches for resolving genomic discrepancies in cancer: a systematic review and clinical perspective
Source: Brief Bioinform. 2025 Nov 2;26(6):bbaf541. doi: 10.1093/bib/bbaf541 (PMC12579925; doi:10.1093/bib/bbaf541)
Supplement: Supplementary_Table_1_bbaf541 [file supplementary_table_1_bbaf541.docx]

**Supplementary Table 1:** Tabular Representation of Applied Methodologies and Key Metrics in Genomic Research

|  | **Author/year** | **Title** | **Dataset** | **Dataset Description** | **Parameters** | **Algorithm/**  **Methodology** | **Feature Extraction Technique/ preprocessing** | **Performance Metrics/ Results** |
| --- | --- | --- | --- | --- | --- | --- | --- | --- |
| 1 | Zodwa Dlamini et al. 2020 | AI and big Data in cancer and Precision Oncology | NGS, WGS, and RNA sequencing | Specific data from The Cancer Genome Atlas (TCGA) and RNA sequencing from tumor samples, blood samples, and liquid biopsies were utilized. | Genetic variability, gene expression profiles, cancer biomarkers, copy number variations (CNVs), and mutational signatures. | ML, AI tools, DL approaches applied to medical imaging and genomic data | Alignment of reads, and feature selection using computational tools for detecting splice variants, CNVs, and pathogenic mutations. | The article highlights AI's role in precision oncology using diagrams to show its flow in diagnostics and integration with NGS and imaging, tables for sequencing platform specifications, and illustrations to depict the AI-driven workflow in genomics and cancer care. |
| 2 | Yang Guo et al. 2018 | Identification of cancer subtypes by integrating multiple types of transcriptomics data with DL in breast cancer | TCGA breast cancer data | The dataset consists of gene expression profiles and transcriptome alternative splicing profiles related to breast invasive carcinoma (BRCA). | The main parameters include gene expression levels and alternative splicing events, which are critical for identifying cancer subtypes. | A hierarchical DL framework called HI-SAE, which works a stacked autoencoder (SAE) neural network to learn high-level demonstrations | Stacked autoencoders to learn representations, k-means clustering approach | The paper presents its results through flowcharts and network diagrams of the hierarchical DL framework; tables comparing SAE configurations and integration methods with p-values; Kaplan-Meier curves for survival probabilities of cancer subtypes; and boxplots of gene expression distributions for specific splicing factors among these subtypes. |
| 3 | Bingsheng He et al. 2020 | An ML framework to trace tumor tissue-of-origin of 13 types of cancer based on DNA somatic mutation | 13 types of cancers somatic mutation data from (ICGC) | The dataset includes somatic mutation profiles, used to identify the primary site of cancers among 13 different types. | Gene length-normalized somatic mutation sequencing data. | Random Forest, with 10-fold cross-validation used for feature selection and categorization. | Feature abstraction involved preprocessing the mutation data to normalize it by gene length. This preprocessing helped in generating a matrix used for further analysis, ensuring that each gene's mutation count was adjusted for its 6 lengths, thus making the dataset suitable for the Random Forest algorithm. | The paper reports performance metrics and visuals, including an average accuracy of 0.8822 and an F1 score of 0.8886 for classifying tissue-of-origin across 13 cancer types using a 600-gene feature set; a confusion matrix illustrating classification performance and misclassifications; and graphs showing accuracy and F1 scores with varying gene counts, highlighting optimization at 600 genes. |
| 4 | Sanad Aburass et al. 2024 | A Hybrid Machine Learning Model for Classifying Gene Mutations in Cancer Using LSTM, BiLSTM, CNN, GRU, and GloVe | Kaggle’s Personalized Medicine: Redefining Cancer Treatment Dataset | Dataset is an ironic source of clinical evidence (text) and genetic alterations associated with cancer therapy. | Numerous performance metrics used include training accuracy, precision, recall, F1 score, and Mean Squared Error (MSE). | A fusion ensemble model that integrates LSTM, BiLSTM, CNN, GRU, and GloVe embeddings for classifying gene mutations. | An embedding matrix was prepared using GloVe embeddings for initial input to the models. | The paper employs various metrics and visualization methods, reporting accuracy, precision, recall, F1 score, and mean squared error (MSE) for model evaluation; comparison diagrams for training and validation metrics, and illustrative figures depicting the architecture and workflow of the hybrid ensemble model. |
| 5 | Bhavneet Bhinder et al. 2021 | AI in Cancer Research and Precision Medicine | TCGA and CAMELYON16 and tumor samples using high-throughput platforms. | The TCGA dataset includes detailed genomic profiles, while other datasets feature DNA and RNA sequencing and high-throughput technologies for functional genomic analyses. | Accuracy, AUC, precision, recall, and F1 scores | CNNs, autoencoder-based methods | Features are automatically learned through DL models from structured data genomic profiles. Preprocessing steps involve normalization and augmentation of data | The paper offers an overview of AI applications in cancer research, using metrics such as accuracy, AUC, and F1 scores for early cancer detection and mutation analysis; flowcharts outlining workflows for AI model development, omics data integration, and model evaluation; and diagrams illustrating relationships between machine learning techniques and applications in cancer research. |
| 6 | Yuhang Guo et al. 2021 | DNA-GCN: Graph Convolutional Networks for Predicting DNA-Protein Binding | The model was evaluated on 50 datasets from the ENCODE project. | Each dataset relates to a specific DNA-binding protein. its positive samples are 101bp DNA sequences experimentally confirmed to bind to this protein.  Negative samples were created by shuffling these positive samples. | Parameters include the settings for the Graph Convolutional Networks (GCN), such as the weights of edges between nodes in the sequence k-Mer graph. | DNA-GCN, a novel method based on GCN | The initial features are derived from constructing a sequence k-Mer graph, where nodes signify sequences and k-Mer’s, and edges are weighted based on k-Mer co-occurrence and sequence relationship. The initial node representation is set as a one-hot encoding. | The paper evaluates model performance using several methods: the area under the ROC curve, AUC as the main metric for DNA-protein binding predictions; architecture diagrams of the DNA-GCN model showing the sequence k-Mer graph structure; and illustrative figures depicting the k-Mer graph construction and information propagation through graph convolution layers. |
| 7 | Yuchen Yuan et al. 2018 | DeepGene: an advanced cancer-type classifier based on deep learning and somatic point mutations | Multiple datasets including BRONCO, ClinVar, COSMIC, and PharmGKB. | BRONCO is a dataset of over 400 mutations linked to genes, diseases, drugs, and cell lines, while ClinVar and COSMIC provide mutation-gene mapping data, and PharmGKB offers mutation-drug triplets. | Several features and parameters were used for machine learning models, including distance and frequency scores, BEST search engine scores, and word vectors constructed from PubMed data. | The use of deep CNNs combined with feature construction techniques like BEST (Biomedical Entity Search Tool) | The preprocessing involved using the BEST tool for feature extraction and the construction of word vectors from PubMed data to provide context and background knowledge for the CNN models. Additionally, the BEST tool was used to obtain search engine scores as features for the classification tasks. | Performance evaluation relies on F1-score and 10-fold cross-validation accuracy. includes flowcharts of the DeepGene workflow, 3D bars plots for parameter sensitivity analysis, and comparison graphs of testing accuracies among different classification models. |
| 8 | Yuchen Yuan et al. 2021 | Cancer-type prediction based on copy number aberration and chromatin 3D structure with convolutional neural networks | GDSC, CCLE, CTRP, PDX, TCGA | These datasets were used for training, cross-validation, and external validation of the model. They encompass multi-omics data such as gene expression, copy number aberration, and mutation data, grouped with drug response data. | Parameters include the number of cell lines, drugs, and sample sizes for resistant and sensitive samples, utilized in training and validation. | Triplet loss (Super. FELT) and binary classification of drug response | The feature selection stage employs a variance threshold based on the elbow method. The feature encoding stage uses different encoders trained with a supervised method, specifically a triplet loss function, aimed at reducing the large dimensionality of omics data and improving the efficiency of the encoder. | The model's performance is evaluated using 10-fold cross-validation accuracy, with comparison graphs of model configurations, architecture diagrams for 1D and 2D CNNs, and accuracy plots comparing DeepCNA to other classification models like SVM, KNN, and Naive Bayes. |
| 9 | Peng-Chan Lin et al. 2018 | Sequential and co-occurring DNA damage response genetic mutations impact survival in stage III colorectal cancer patients receiving adjuvant oxaliplatin-based chemotherapy | The dataset is from TCGA | The dataset comprised molecular data such as DNA copy number variations, RNA-seq gene expression, and somatic mutation data for oligodendrogliomas. This data was used to stratify these brain tumors into clinically relevant molecular subtypes. | Parameters mentioned include gene-specific copy number log ratios and the use of hierarchical clustering to identify molecular subgroups within oligodendrogliomas. | Hierarchical clustering | The feature extraction involved determining gene-specific copy number log ratios for each oligodendroglioma based on its DNA copy number profile. Additionally, statistical methods like hierarchical clustering were used to classify these tumors into distinct molecular subgroups. | The analysis includes Kaplan-Meier survival curves showing survival probabilities for genetic mutation subgroups; AUC values from time-dependent ROC curves to assess predictive efficacy for survival over time; decision trees for survival prediction based on mutation sequences; and heatmaps with hierarchical clustering to illustrate somatic mutations and risk groups based on clinical characteristics. |
| 10 | Irina Kalatskaya et al. 2021 | ISOWN: Accurate Somatic Mutation Identification in the Absence of Normal Tissue Controls | The dataset is from the National Cheng Kung University Hospital (NCKUH). | The study collected clinical information and tumor-targeted deep sequencing data. Tumor tissues and blood samples were collected at the time of enrollment. The sequencing targeted known cancer-related genes to study the impact of DNA damage response genetic mutations on survival outcomes. | In this analyzed the order and co-occurrence of somatic mutations and their links to recurrence-free survival, using statistical parameters like hazard ratios, p-values, and AUC for predictive efficacy. | The methodology included targeted tumor sequencing with Oncomine Comprehensive Assays, reconstruction of phylogenetic trees to study evolution, and statistical analyses using chi-square tests, Fisher’s exact tests, and Kaplan-Meier curves with Cox models. Machine learning models, specifically recursive partitioning (RPA) and LASSO were used for survival analysis and feature selection. | Feature extraction was performed using targeted deep sequencing data, aligning reads to the hg19 reference genome and calling variants with the Torrent Variant Caller. The study also used phylogenetic tree construction for evolutionary trajectory analysis and applied the LASSO algorithm for feature selection in survival analysis. | The model achieved an F1-score ranging from 75.9% to 98.6% across various cancer types based on mutation load and dataset; AUC was used in cross-validation to assess performance; tenfold cross-validation validated the model with TCGA and external datasets. The visuals include workflow illustrations of the ISOWN framework, performance comparison graphs for different machine learning classifiers, and Kaplan-Meier survival plots with bar charts for mutation statistics. |
| 11 | Michaela Unger et al. 2017 | Deep learning in cancer genomics and histopathology | TCGA dataset including 6 different cancer types and approximately 1600 samples | The study used both deep-targeted and whole-exome sequencing data. It included various cancer types and different tissue preparations, with a detailed focus on distinguishing somatic mutations in the absence of normal tissue. | Parameters include the classification of variants into somatic and germline categories, focusing on their accuracy and misclassification rates. The algorithm's performance was evaluated using metrics like F1-measure, accuracy, and the area under the ROC curve. | ISOWN, a machine learning algorithm, was used to distinguish somatic mutations without matched normal samples, employing supervised learning techniques and evaluated using tenfold cross-validation. | Features included annotations against databases like COSMIC, dbSNP, and PolyPhen-2, using variant allele frequency, sample frequency, and mutation impact predictions to train the classification model. | The evaluation methods include AUROC for model performance in cancer subtype prediction, C-index for accuracy in survival prediction, and diagrams showing AI applications in histopathology, along with timelines of deep learning advancements in cancer genomics. |
| 12 | Sayed Mohammad Ebrahim Sahraeian et al. 2024 | Deep convolutional neural networks for accurate somatic mutation detection | The dataset includes The Cancer Genome Atlas (TCGA). Data including genomic and histopathological images. | The article discusses the integration of histopathological image data and genomic data, reviewing various studies that have utilized these types of data to improve cancer diagnostics and prognostics. | Parameters studied included diagnostic accuracy, cancer subtyping, prediction of survival outcomes, detection of genetic alterations, and response to treatments using deep learning techniques on combined datasets of histopathology and genomic data. | Deep learning methodologies are applied in the analysis of cancer, focusing on the integration of multimodal data (histopathology and genomics) to improve predictive models in oncology. | Techniques include the use of convolutional neural networks (CNNs) for image data and various normalization and transformation techniques for genomic data to prepare inputs for deep learning models. | The study achieved F1-scores of 99.6% for SNVs and 97.2% for INDELs in synthetic datasets, evaluated NeuSomatic's accuracy using precision-recall curves, and conducted extensive cross-validation across various datasets and tumor purity scenarios, supported by workflow diagrams and performance graphs comparing F1-scores and precision-recall curves. |
| 13 | Hongjian Qi et al. 2021 | MVP predicts the pathogenicity of missense variants by deep learning | The Cancer Genome Atlas (TCGA) and the Ivy GAP dataset. | TCGA was used for correlation analysis of segmented tumor regions with specific RNA glioma datasets, while Ivy GAP consists of 805 whole-slide images of brain tumors from 32 patients for training a deep convolutional neural network for semantic segmentation. | The study focuses on the correlation of segmented tumor regions with genetic signatures and survival rates, exploring how these regions contribute to poor prognosis in glioblastoma patients. | The model’s name is deep convolutional neural network (DCNN) | Preprocessing: Images were resized, and patches were extracted at different scales for training the DCNN. A pixel-normalization method was used during preprocessing to scale pixel values to the range [0,1].  Feature Extraction: The DCNN used layers designed for semantic segmentation to process and extract features from the WSIs, aiding in the identification and analysis of different tumor regions | The model achieved high AUC values (0.99 for constrained genes and 0.97 for non-constrained genes) in cross-validation, used precision-recall curves for pathogenicity prediction, and ROC curves for performance comparison, with diagrams showing the MVP model's workflow and predictive performance against existing methods. |
| 14 | Peter Peneder et al. 2021 | Multimodal analysis of cell-free DNA whole-genome sequencing for pediatric cancers with low mutational burden | HGMD, UniProt, ClinVar, DiscovEHR, and VariBench | The datasets consist of curated sets of pathogenic and benign variants used for training and testing a model to predict the pathogenicity of missense variants. This includes rare and common variants, functional annotations, and protein interaction data. | The model uses various parameters including gene mutation intolerance metrics, conservation scores, amino acid constraint scores, and deleteriousness scores from other methods. | The methodology is based on a deep residual neural network (ResNet) model, trained separately on datasets of constrained and non-constrained genes to predict the pathogenicity of missense variants. | Features used include local context features, gene mutation intolerance, protein structure and modifications, and scores from previous prediction methods. Preprocessing involves normalizing scores using rank percentiles and managing missing values. | The article evaluates output using ROC curves for classification performance, AUC for accuracy, fragment-size distribution histograms for DNA fragmentation, box plots and heatmaps for data visualization, and correlation plots to show relationships between cfDNA fragmentation and clinical metrics. |
| 15 | Muta Tah Hira et al. 2021 | Integrated Multi-Omics Analysis of Ovarian Cancer Using Variational Autoencoders | Genomics of Drug Sensitivity in Cancer (GDSC) and Cancer Cell Line Encyclopedia (CCLE) | The datasets include gene expression, somatic mutation, and copy number variation data for 319 cancer cell lines (GDSC) and 478 cancer cell lines (CCLE). The datasets are used to predict drug sensitivity in cancer cell lines, incorporating data on drug structures and cellular genomic features. | Parameters include log-transformed IC50 values, chemical structure molecular fingerprints, pathway enrichment scores, and genomic features like mutations and copy number variations. | The article introduces a model called PathDSP, which uses a deep neural network to integrate pathway-based features with chemical structure information for predicting drug sensitivity. | Feature extraction includes using gene expression data to calculate pathway enrichment scores using the ssGSEA algorithm, mutation and copy number variation data for network-based pathway enrichment analysis, and molecular fingerprints from chemical structures. Data preprocessing involves normalization and handling of missing values for robust model training. | The analysis includes clustering accuracy shown in scatter plots for 2D and 3D data embeddings, classification metrics such as accuracy, precision, recall, F1 score, and confusion matrices, survival analysis using Kaplan-Meier curves, C-index, Cox model p-values, and Brier scores, as well as feature importance illustrated through correlation analysis and heatmaps linking latent features to genomic data. |
| 16 | Cheng-Hong Yang et al. 2021 | Applications of Deep Learning and Fuzzy Systems to Detect Cancer Mortality in Next-Generation Genomic Data | The study utilized TCGA datasets, specifically focusing on ovarian cancer. Mono-omics and multi-omics (di-omics and tri-omics) data. | The dataset contains genomic, transcriptomic, and epigenomic data for ovarian cancer. Features include mRNA, CNV/CNA, DNA methylation, RNA seq, and miRNA across various sample sizes. | The study discusses the handling of high-dimensional omics data, focusing on input feature dimensions and sample sizes. | The methodology is centered on Variational Autoencoders (VAE) and Maximum Mean Discrepancy VAE (MMD-VAE). These models are used for dimensionality reduction, clustering, and survival analysis. | The article details preprocessing steps like normalization using min-max techniques, removing missing values, and dataset integration. These steps ensure that multi-omics features are prepared and compatible for further analysis. | The analysis includes accuracy tests for model performance, Kaplan-Meier survival curves for survival analysis, forest plots for hazard ratios from CoxPH regression, mosaic plots for clinical feature distributions, and CIRCOS plots for visualizing candidate genes and mutation variants. |
| 17 | **Feixiong Cheng et al.** 2021 | Advances in computational approaches for prioritizing driver mutations and significantly mutated genes in cancer genomes | Haploinsufficient genes (HIS genes) and haploinsufficient genes (HS genes) | The datasets include 287 HIS genes and 574 HS genes selected from a copy number variation study of 2026 normal individuals. These genes are linked to various human diseases and biological processes, particularly cancers. | The study primarily deals with genomic and epigenomic patterns that indicate haploinsufficiency in genes, focusing on mutations and gene dosage sensitivities. | The methodology employed is a deep forest algorithm named HaForest, which incorporates multiscale scanning and a cascade forest structure to identify haploinsufficient genes from epigenomic data. | Epigenomic data were processed using promoter and enhancer features extracted from predefined topologically associated domains. Features were consolidated into a matrix and then passed through the LightGBM library to refine feature selection before input into the HaForest model. | The paper uses computational tools and frameworks to prioritize driver mutations and significantly mutated genes (SMGs), with Figure 1 illustrating the computational approaches for identifying these mutations in cancer genomes and tables summarizing data resources and tools, including Table 1 for data resources and Table 2 for computational tools. |
| 18 | Amena Mahmoud et al. 2016 | Breast Cancer Survival Prediction Modeling Based on Genomic Data: An Improved Prognosis-Driven Deep Learning Approach | COSMIC and TCGA data on over 10,000 specimens from 25+ tumor types in cancer genomes. | COSMIC (Catalogue of Somatic Mutations in Cancer) contains millions of coding point mutations. TCGA (The Cancer Genome Atlas) and ICGC (International Cancer Genome Consortium) offer comprehensive genomic, epigenomic, and transcriptomic profiles of various cancer genomes. | The parameters discussed focus on the identification of driver mutations, significant mutated genes (SMGs), and the utilization of next-generation sequencing (NGS) data. | The model names are mutation frequency-based approaches, functional impact-based approaches, structural genomics-based approaches, network or pathway-based approaches, and data integration-based approaches. | Techniques mentioned include the analysis of protein 3D structures, functional impacts of mutations, and integration of genomic data with pathway and network data. | The evaluation includes accuracy, precision, recall, and F1 score metrics, confusion matrices, accuracy/loss curves for model performance, and a comparison showing the LSTM model achieved the highest accuracy at 98.7%. |
| 19 | Runpu Chen et al. 2020 | Deep-learning approach to identifying cancer subtypes using high-dimensional genomic data | METABRIC (Molecular Taxonomy of Breast Cancer International Consortium) | The METABRIC dataset contains expression profiles of 25,160 genes from 1,989 primary breast tumor samples and 144 normal breast tissue samples. For analysis, the authors retained the top 20,000 most variant genes. | Parameters such as the number of clusters (K), the trade-off parameter (α), and the regularization parameter (k) were estimated during the model optimization. The dataset was split into training (80%) and testing (20%) sets. | DeepType is a deep learning-based approach that a multi-layer neural network for representation learning and combines classification loss, and clustering loss, and integrates joint supervised classification and unsupervised clustering for identifying cancer subtypes. | The preprocessing included retaining the top 20,000 variant genes. Additionally, the deep learning model employed a feature selection mechanism through regularization to identify and focus on the most relevant genes for subtyping. | The analysis includes Silhouette Width for clustering quality, Davies-Bouldin Index for cluster separation, Average Purity and Normalized Mutual Information for alignment with clinical variables, t-SNE plots for visualizing cancer subtypes, and heatmaps to display gene expression patterns among subtypes. |
| 20 | Hao Zhang et al. 2023 | DeepFilter: A Deep Learning Based Variant Filter for VarDict | The paper uses three main datasets: SEQC-II, Genome in a Bottle (GIAB) samples, and BamSurgen synthetic data. | The SEQC-II dataset contains tumor-normal paired samples, the GIAB dataset combines two samples to simulate somatic mutation detection, and the synthetic dataset includes 500,000 SNVs and 400,000 InDels generated in silico. | The model was trained in PyTorch with a learning rate of 0.001, and hidden layer sizes of 80-170 neurons for SNVs and InDels, using the Adam optimizer and dropout to prevent overfitting. | DeepFilter is a deep learning-based variant filter for VarDict. It uses a multi-layer perceptron (MLP) neural network to classify variants as true or false positives. Two separate models were developed for SNVs and InDels, trained on tumor-normal paired data. | Forty-five features for InDels and forty-two features for SNVs were selected from VarDict's output, including allele frequency, mapping quality, and base quality, with features standardized and categorical features one-hot encoded while numerical features were normalized for the neural network. | The article uses the F1-score, precision, recall, false positive filtration rate (FFR), and true positive retention rate (TRR) to evaluate output performance. It also includes ROC curves to showcase model performance, with the area under the curve (AUC) values provided for SNV and InDel models. |
| 21 | Matteo Bastico et al. 2023 | DrOGA: An Artificial Intelligence Solution for Driver-Status Prediction of Genomic Mutations in Precision Cancer Medicine | Cancer Genome Interpreter (CGI), FASMIC, dbSNP, and IntOGen. | The combined dataset consists of 16,360 driver samples and 46,444 neutral (passenger) mutations, making a total of 62,804 variants. The data sources include somatic mutations linked to cancer, particularly focusing on non-synonymous variants (e.g., missense mutations). | The optimization of hyperparameters such as learning rates, loss functions (like binary cross-entropy), and regularization methods using the Adaptive eXperimentation (AX) algorithm, employing both classical machine learning models (SVM, Random Forest, XGBoost) and deep learning models (CNN, Multi-layer Perceptrons). | The framework name is Driver-Oriented Genomics Analysis (DrOGA), which employs machine learning and deep learning techniques to classify driver versus neutral mutations, utilizing models like CNN and XGBoost for optimization, and incorporates eXplainable Artificial Intelligence (XAI) to interpret model outputs to clinical annotations. | The dataset is processed with 70 features derived from ANNOVAR annotations. These features include pathogenicity predictors (e.g., MetaSVM, REVEL), conservation scores (e.g., GERP++, PhyloP), and functional predictors (e.g., SIFT, PolyPhen2). The dataset was further cleansed and normalized to ensure high-quality inputs for the models. Techniques such as rank scoring, one-hot encoding, and min-max normalization were applied. | The paper uses various metrics, including F1-score, precision, recall, ROC curves, and area under the curve (AUC) values to evaluate the classification performance. It also includes SHAP values for explainability in feature importance analysis. |
| 22 | Jie Peng et al. 2022 | Deep learning to estimate durable clinical benefit and prognosis from patients with non-small cell lung cancer treated with PD-1/PD-L1 blockade | Peripheral blood samples or tumor tissues from non-small cell lung cancer (NSCLC) patients. | The study involved whole-exome sequencing (WES) or next-generation sequencing (NGS) data, combined with clinical outcomes related to PD-1/PD-L1 blockade therapy. | CNN alongside conventional machine learning models like Support Vector Machine (SVM) and Random Forest (RF) to predict durable clinical benefit (DCB) and progression-free survival (PFS) in NSCLC patients undergoing immunotherapy. | CNN trained on single nucleotide variant (SNV) data from WES or NGS to predict immunotherapy outcomes and outperforming conventional machine learning models like SVM and Random Forest in predicting durable clinical benefit and prognosis. | Genomic features, including 55 somatic mutations, were selected using a Random Forest (RF) algorithm from WES and NGS data, which included key mutational signatures and tumor mutational burden (TMB) metrics. | The study uses metrics such as F1-score, ROC curves, AUC values, and Kaplan-Meier survival analysis for progression-free survival (PFS) and overall survival (OS) to assess predictive performance. It also includes a nomogram based on combined models (CNN, SVM, and RF) for prognosis estimation. |
| 23 | Navodini Wijethilake et al. 2021 | Glioma Survival Analysis Empowered with Data Engineering—A Survey | The Cancer Genome Atlas, the Chinese Glioma Genome Atlas, and other public sources like Gene Expression Omnibus (GEO). | The datasets include **whole-exome sequencing (WES)** data, gene expression profiles, mutation profiles, methylation data, and imaging data (MRI scans). These datasets provide a comprehensive view of molecular and imaging features for glioma patients, which are used for survival analysis and prognosis prediction. | The study uses different machine learning and deep learning models, such as CNNs, SVMs, RSFs, and ANNs. These models predict glioma patient survival using radiomic, genomic, and clinical data. | The authors investigate various ML and DL techniques for predicting survival by integrating radiomic and genomic data, using feature extraction methods like PCA and recursive feature elimination on MRI and genomic data, while also applying Cox models and Kaplan-Meier analysis for traditional statistical evaluation. | The study uses preprocessing techniques such as normalization, dimensionality reduction (PCA), and feature selection, applies MRI segmentation to extract tumor regions and obtain texture, shape, and intensity features, and derives genomic features from whole-exome sequencing and gene expression data, including mutation profiles and methylation signatures. | The study uses Kaplan-Meier curves, Cox proportional hazard models, decision trees, and survival trees for survival prediction. It also discusses various machine learning and statistical methods for analyzing glioma patient survival. |
| 24 | Pooya Mobadersany et al. 2018 | Predicting Cancer Outcomes from Histology and Genomics Using Convolutional Networks | 1: TCGA Lower Grade Glioma (LGG) cohort.  2: TCGA Glioblastoma (GBM) cohort | The dataset included H&E-stained whole-slide images (WSIs) from formalin-fixed paraffin-embedded glioma specimens. Regions of interest (ROIs) were manually identified for each slide to contain viable tumor tissue for analysis. The genomic data included IDH mutation status and 1p/19q codeletion status, which are molecular biomarkers used for glioma classification. | The study measured the concordance between predicted risks and actual survival using Harrell’s c-index, a statistic that evaluates the accuracy of time-to-event predictions. It also compared the deep learning model's performance against traditional histologic grading and molecular subtyping. | The study presents a Survival Convolutional Neural Network (SCNN) that integrates histology images and genomic biomarkers to predict time-to-event outcomes, combining convolutional layers for image processing with a Cox model, while the genomic survival convolutional neural network (GSCNN) further incorporates genomic variables like IDH mutation and 1p/19q codeletion. | Histology images were preprocessed by manually selecting regions of interest (ROIs), cropping to high-power fields (HPFs), and applying color normalization and data augmentation techniques, while genomic data like IDH mutation status and 1p/19q codeletion were incorporated into the deep learning model's fully connected layers. | This study evaluates predictive accuracy using Harrell’s C-index, Kaplan-Meier survival curves, Cox proportional hazards models, and heat maps to visualize histological features associated with risk. It combines histology with genomic data for improved outcome prediction. |
| 25 | Hui Y. Xiong et al. 2015 | The Human Splicing Code Reveals New Insights into the Genetic Determinants of Disease | 1: NCBI GSE30611  2: datasets linked to spinal muscular atrophy, nonpolyposis colorectal cancer (Lynch syndrome), and autism spectrum disorder (ASD). | The study mapped thousands of SNVs and their impact on RNA splicing regulation across various tissues. By modeling exon inclusion rates (Ψ), the authors identified genetic variants that cause mis regulation of splicing, which contributes to various diseases, including cancer and neurological disorders. | The study employed metrics such as percent spliced-in (Ψ) for RNA splicing and regulatory scores for assessing SNV impact on splicing regulation, while prediction accuracy was evaluated using AUC (area under the ROC curve) and correlation with experimental results. | 1: Computational machine learning model  2: Deep learning model | 1: Features were extracted from genomic regions surrounding exons, including counts of splicing factor motifs, intron/exon lengths, 1-3mer frequencies, and RNA-binding protein affinities.  2: RNA-seq data was used to estimate exon inclusion (Ψ) in 16 tissues, and SNVs were computationally scored based on their predicted impact on splicing. | The paper evaluates its computational model's accuracy using metrics such as the area under the ROC curve (AUC), with specific values like 95.5% AUC for high inclusion versus low inclusion classification tasks. Visualizations include ROC curves, bar charts, and correlation plots. The study also uses heatmaps to illustrate the effect of specific splicing code predictions and plots comparing predictions to experimental data. |
| 26 | Richard J. Chen et al. 2022 | Pathomic Fusion: An Integrated Framework for Fusing Histopathology and Genomic Features for Cancer Diagnosis and Prognosis | 1: TCGA Glioma dataset (TCGA-GBMLGG)  2: TCGA Clear Cell Renal Cell Carcinoma dataset (TCGA-KIRC) | The datasets consist of multimodal data, including histopathology images and genomic features (CNVs, mutations, and RNA-Seq gene expression) from cancer patients, which are used for survival outcome prediction and cancer grading. | Survival outcome prediction is evaluated using the concordance index (C-index), which measures the fraction of correctly ordered pairs of samples based on survival times.  Grade classification is evaluated using metrics such as AUC (Area Under the Curve), average precision, and F1-score. | The model names used in this study are these  1: Pathomic Fusion  2: Convolutional Neural Networks (CNNs)  3: Graph Convolutional Networks (GCNs)  4: Self-Normalizing Networks (SNNs) | 1: For histopathology images, features are extracted using CNNs from 512×512 regions of interest at 20× magnification.  2: Cell graphs are constructed using K-Nearest Neighbors (KNN) to define cell-cell interactions, and features like cell contour and texture are manually extracted along with unsupervised features via contrastive predictive coding.  3: Genomic features (CNV, mutation status, RNA-Seq) are processed with SNNs to handle the high-dimensional nature of genomic data and prevent overfitting. | The study uses various metrics, including the Concordance Index (C-index), Area Under the Curve (AUC), Average Precision (AP), F1-score, and Kaplan-Meier survival curves. It also includes ROC curves and hazard distribution plots for survival prediction. The use of multimodal interpretability methods, like Integrated Gradients and Grad-CAM, provides insights into how the model utilizes both histological and genomic features for predictions. |
| 27 | Richard J. Chen et al. 2022 | Pan-cancer integrative histology-genomic analysis via multimodal deep learning | TCGA which includes 6,592 WSIs and molecular profile data from 5,720 patient samples across 14 cancer types. | The dataset includes digitized high-resolution histology slides (H&E) and molecular profile features, such as **mutation status, copy number variation, and RNA sequencing (RNA-seq) expression**. | The main parameters involve **predictive performance metrics** like concordance index (c-Index) and survival AUC, used to evaluate the effectiveness of the prognostic models. | The proposed multimodal fusion deep learning model (MMF) combines an attention-based network for histopathology with a self-normalizing network for molecular data, using a Kronecker product to model interactions between these features. | Features were extracted from both WSIs and molecular data, with **weakly supervised learning** used for survival-outcome prediction. The study also applied attention- and attribution-based interpretability techniques to identify key prognostic features. | The study uses several metrics to evaluate model performance, including the Concordance Index (C-index), Kaplan-Meier survival curves, ROC curves, and survival AUC. Visualizations include attention-based heatmaps, attribution-based interpretability maps for genomic features, and Kaplan-Meier curves for survival outcomes. Additionally, the Pathology-Omics Research Platform for Integrative Survival Estimation (PORPOISE) provides an interactive interface for further exploring model predictions and feature attributions. |
| 28 | Cong Wang et al. 2024 | PLANNER: A Multi-Scale Deep Language Model for the Origins of Replication Site Prediction | The dataset is sourced from the **DeOri database**, a public repository of eukaryotic origins of replication (ORIs). | The dataset contains experimentally confirmed ORIs of four eukaryotic organisms: Homo sapiens, Mus musculus, Drosophila melanogaster, and Arabidopsis thaliana. Positive samples were sub-sequences of 300 base pairs (bp) long, extracted from confirmed ORIs. Negative samples were created by taking sequences upstream and downstream from the ORIs. The CD-HIT tool was used to remove samples with more than 80% pairwise sequence identity to avoid redundancy. | Key performance metrics include **AUC (Area Under the ROC Curve), ACC (Accuracy), MCC (Matthews Correlation Coefficient),** and **F1 score**, which were used to evaluate prediction performance. | The paper introduces a deep learning model called **PLANNER** (DeeP LeArNiNg prEdictor for ORI). PLANNER is based on the **DNABERT** model, a pre-trained transformer architecture that uses DNA k-Mer’s. The approach utilizes multi-scale feature extraction and combines ensemble learning strategies, including voting and blending methods, to enhance prediction robustness. | 1: multi-scale information processing is employed using k-Mer’s of different sizes (3-mers, 4-mers, 5-mers, and 6-mers).  2: Sequences are tokenized into k-tuples, and DNABERT models are fine-tuned for species-specific predictions.  3: The models are trained using cross-validation and ensemble learning to ensure accurate ORI predictions across multiple species and cell types. | The study evaluates PLANNER using metrics such as accuracy (ACC), Matthews Correlation Coefficient (MCC), Area Under the Curve (AUC), and F1-score. The study includes visualizations like ROC curves, ablation study plots (to assess the impact of multi-scale k-Mer’s), and heatmaps for attention maps, highlighting the most informative sequence regions for origin of replication (ORI) prediction. |
| 29 | Shashank Singh et al. 2019 | Predicting Enhancer-Promoter Interaction from Genomic Sequence with Deep Neural Networks | Datasets used  1: GM12878  2: HeLa-S3  3: HUVEC  4: IMR90  5: K562  6: NHEK | The dataset consists of enhancer-promoter pairs, labeled as interacting (positive) or non-interacting (negative), based on high-resolution chromatin contact data from Hi-C experiments. Positive pairs were augmented by adding flanking sequences. | The cell line-specific enhancer-promoter interactions were predicted using deep neural networks. Various performance metrics were evaluated, such as AUROC (Area Under ROC), AUPR (Area Under Precision-Recall), and F1 score. | The authors developed the SPEID model, a deep learning framework with convolutional layers for extracting genomic patterns, an LSTM layer for capturing long-range dependencies, and a dense layer for predicting enhancer-promoter interactions. | Feature extraction in this model is fully automatic through deep learning. The study uses convolutional filters to learn sequence motifs, while data augmentation is used to extend enhancers and promoters to fixed-length sequences for input into the model. | The study evaluates the predictive performance of its model using AUROC (Area Under the Receiver Operating Characteristic Curve), AUPR (Area Under the Precision-Recall Curve), and F1-score. Additionally, it uses ROC curves and precision-recall curves to visually demonstrate model performance across different cell types. Heatmaps are also used to display important sequence features for enhancer-promoter interactions. |
| 30 | Jia Xu et al. 2019 | Translating Cancer Genomics into Precision Medicine with Artificial Intelligence: Applications, Challenges, and Future Perspectives | Discussed datasets  1: TCGA  2: COSMIC  3: ClinVar | Multi-omics data, including DNA-seq, RNA-seq, proteome, and epigenome, with focus on genomics for cancer research. Specific datasets like TCGA offer genotype-phenotype data for analyzing cancer progression and treatment. | The parameters vary depending on the task, including tumor mutational burden (TMB), molecular subtypes, sequencing depth, allele frequency, quality scores, etc. These parameters are related to the assessment of genomic variants and their implications in cancer treatment. | The paper focuses on the use of **machine learning** and **deep learning algorithms** for variant calling, variant interpretation, and cancer diagnostics. Techniques like **convolutional neural networks (CNNs), Random Forests,** and **deep neural networks** are highlighted. | Feature extraction is discussed primarily in terms of **NGS data preprocessing** and variant calling. It includes steps like quality evaluation, recalibration, indel realignment, alignment to reference genomes, and data normalization for integrative analyses. | This article reviews AI applications in cancer genomics without focusing on specific model evaluation metrics like F1 scores or ROC curves. Instead, it discusses the integration of machine learning and deep learning into workflows for cancer genomics, outlining both benefits and challenges. Figures include schematic illustrations of AI-driven workflows in genomics and conceptual representations of machine learning processes applied to cancer diagnostics and variant interpretation. |
| 31 | Carlos H. M. Rodrigues et al. 2024 | Exploring the Effects of Missense Mutations on Protein Thermodynamics through Structure-Based Approaches: Findings from the CAGI6 Challenges | **COSMIC database** for selecting missense variants of MAPK1 and MAPK3. These were evaluated as part of the CAGI6 challenge. | 1: MAPK1 and MAPK3: Missense variants from COSMIC were experimentally evaluated using Gibbs Free Energy of Folding (ΔΔG) values for both phosphorylated and unphosphorylated forms of these proteins.  2: Calmodulin: 16point mutations were assessed for their stability and unfolding under thermal denaturation conditions. | Key parameters include ΔΔG for protein stability, ΔS for mutation-induced flexibility, AUC for model performance evaluation, and RMSE for predictive error of ΔΔG values. | Use models in this article are ENCoM, mCSM, DUET, DynaMut, DynaMut2, and DDMut | The features were extracted based on: Protein structures (PDB entries) for the mapping of mutations. The use of physicochemical properties, distance pattern signatures, and solvent accessibility parameters in their machine learning models. | The study measures prediction accuracy using AUC (Area Under the ROC Curve), correlation coefficients (Pearson, Kendall, Spearman), and Root Mean Square Error (RMSE) for evaluating protein stability changes due to missense mutations. Visualizations include ROC plots for the classification of stabilizing vs. destabilizing mutations, as well as heatmaps to demonstrate protein stability changes. |
| 32 | Yoshifumi Shimada et al. 2021 | Histopathological Characteristics and Artificial Intelligence for Predicting Tumor Mutational Burden-High Colorectal Cancer | Datasets are used JP-CRC cohort, TCGA-CRC cohort | JP-CRC cohort: This cohort included patients who underwent primary tumor resection and were tested for TMB-H status using gene panel testing.  TCGA-CRC cohort: CRC patients from TCGA whose histopathological images were downloaded and used for training and validation in a CNN-based model. | Tumor-infiltrating lymphocytes (TILs) over 10 per 5 high-power fields predict TMB-H CRC; the CNN model achieved an AUC of 0.934 for TMB-H prediction, and the TMB-H Index is the ratio of TMB-H tiles to total neoplastic tiles. | InceptionV3 (InceptionV3) was developed to predict TMB-H CRC directly from H&E-stained images. | The H&E-stained slide images were divided into square tiles (300x300 pixels), and tiles with over 80% neoplastic tissue were labeled as "neoplastic."  The colors of the tiles were normalized, and transformation of color parameters (HSV) was applied to improve accuracy.  Digital image processing and tile labeling were central preprocessing steps before training the CNN model. | The study used metrics like Area Under the Curve (AUC) for performance evaluation. Key visualizations included ROC curves to evaluate prediction accuracy and heatmaps that highlighted tissue areas most predictive of high tumor mutational burden (TMB-H). The CNN model demonstrated an AUC of 0.910 for predicting TMB-H status based on histopathological characteristics. |
| 33 | Yuchen Yuan et al. 2016 | DeepGene: An Advanced Cancer Type Classifier Based on Deep Learning and Somatic Point Mutations | TCGA-DeepGene dataset.  It includes somatic point mutations from 12 cancer types, with data from 3122 samples and 22,834 genes. | The dataset contains binary mutation data, where each gene is either mutated (1) or not mutated (0) in a given sample. The dataset represents 12 types of cancers, including breast, lung, and colorectal cancers, among others.  The mutation data is processed using methods designed to filter out irrelevant genes and reduce data sparsity. | Mean Rank Ratio (MRR), AUC (Area Under the Curve), and Accuracy are used to assess the performance of the classification models.  Other parameters include the non-zero element threshold (used to reduce data sparsity) and distance thresholds for clustering genes. | The DeepGene model is a deep neural network that employs Clustered Gene Filtering (CGF) for high-mutation gene identification and Indexed Sparsity Reduction (ISR) to reduce data sparsity, followed by a DNN classifier with multiple hidden layers for cancer-type classification. | The paper employs feature extraction via **CGF** to isolate the most relevant gene subsets, and **ISR** to minimize the influence of data sparsity. The final DNN classifier is used for classification, after preprocessing the gene data through these techniques. | The study presents its results primarily through **accuracy** metrics and comparative bar plots. The paper highlights 10-fold cross-validation accuracy improvements over traditional classifiers, visualized in 3D bar plots to demonstrate parameter sensitivity and comparisons against SVM, KNN, and Naive Bayes classifiers. The paper does not report F1 scores or other performance metrics like precision and recall. |
| 34 | Imane Boudellioua et al. 2019 | DeepPVP: Phenotype-Based Prioritization of Causative Variants Using Deep Learning | ClinVar database (31,156 pathogenic variants, 23,808 benign variants); 1000 Genomes Project. | ClinVar dataset: Contains both pathogenic and benign variants associated with specific diseases. Pathogenic variants are linked to diseases in the OMIM database, and benign variants are selected based on their clinical significance in the ClinVar database.  Synthetic exomes: Variants from the 1000 Genomes Project are used to simulate patient exomes by inserting causative variants associated with diseases. | ROC AUC (Receiver Operating Characteristic Area Under Curve) and AUPR (Area Under Precision-Recall Curve) are used to assess the performance of the models.  Metrics such as accuracy, recall at rank one, and recall at rank ten are calculated for evaluation. | DeepPVP is a deep neural network model that integrates pathogenicity and phenotype similarity scores, featuring a five-layer architecture and trained with nested cross-validation for hyperparameter optimization. | Variants are annotated with pathogenicity scores from CADD, DANN, and GWAVA, and phenotypes are extracted from the OMIM and Human Phenotype Ontology (HPO) databases.  The model uses one-hot encoding to represent categorical features like inheritance mode and zygosity.  Missing values are handled through mean imputation, and additional flags for missing values are used as features. | The paper evaluates DeepPVP's performance through **ROC-AUC** (Receiver Operating Characteristic - Area Under Curve) and **AUPR** (Area Under the Precision-Recall Curve) metrics, along with bar charts comparing performance against other methods. The cross-validation results are used to highlight the effectiveness of the model, but F1 scores or other efficiency metrics specific to deep learning model evaluation are not explicitly shown. |
| 35 | Hang Zhang et al. 2019 | VariFAST: A Variant Filter by Automated Scoring Based on Tagged-Signatures | Genome in a Bottle (GIAB); Germline Variant Data from six samples (HG001, HG002, HG003, HG004). | Germline Dataset: Includes high-confidence benchmark variants from multiple sequencing platforms, useful for testing variant calling accuracy.  Somatic Dataset: Focuses on whole exome sequencing data from cancer cases, where somatic mutations are identified and filtered. | The variant score (v-score) combines 16 or 18 metrics to assess false positives, while AUC evaluates model performance, and Precision, Recall, F1-Score, and Matthews Correlation Coefficient (MCC) measure filtering effectiveness. | VariFAST is an automated tool that filters false positives the models used are a weighted scoring system (v-score) for assessing variants and XGBoost for refining variant filtering, integrated with the GATK Best Practices pipeline for high-quality detection. | Variant Metrics: The tool calculates 16 metrics for germline variants and 18 metrics for somatic variants. Metrics include factors like low coverage, variant allele frequency, mismatch rates, and read mapping quality.  Preprocessing: Raw sequencing data are processed to extract variants using standard tools like BWA MEM and GATK Haplotype Caller, followed by variant refinement through the VariFAST pipeline. | The paper utilizes the **Fβ score, ROC curves,** and **Matthews’s correlation coefficient (MCC)** as output metrics to evaluate the performance of the VariFAST approach in filtering false-positive variants from genomic sequencing data. The authors use ROC curves and AUC values to compare the performance of their tool with other models like VQSR (Variant Quality Score Recalibration) and demonstrate significant improvements, particularly for INDEL variant filtering. Bar plots and precision-recall diagrams further illustrate performance. |
| 36 | Martin Palazzo et al. 2019 | A Pan-Cancer Somatic Mutation Embedding Using Autoencoders | International Cancer Genome Consortium (ICGC); somatic mutation data from 11,183 tumor samples across 14 cancer types and 40 tumor subtypes. | The dataset includes simple somatic mutation (SSM) data, with variants classified into deleterious mutations (such as frameshift, missense, and stop-gained mutations) and non-deleterious mutations. The mutational profiles from 11,183 samples are reduced to 12,424 genes after filtering based on mutation occurrence in at least 50 samples, leading to a less sparse dataset. | AUC evaluates the classification performance of tumor subtypes, Kernel Target Alignment assesses the quality of the learned latent space for biological signals, and Mutual Information measures the clustering effectiveness of tumor samples by subtypes. | The model is a multi-modal autoencoder that learns a low-dimensional latent space from somatic mutational profiles using separate encoders for deleterious and non-deleterious mutations, validated with Kernel Learning and classified with one-class SVMs. | Preprocessing involves filtering genes based on mutation frequency and zero-one normalization of the mutation data. The resulting dataset is split into training and test sets, with cross-validation used to tune the model’s hyperparameters.  The autoencoder learns separate representations for deleterious and non-deleterious mutations, which are combined in the latent space. | The study evaluates the performance of its multi-modal autoencoder model using several output metrics, including **AUC-ROC** for tumor subtype classification and **Kernel Target Alignment (KTA)** for assessing clustering quality. Additionally, **mutual information (MI)** scores are used to evaluate clustering consistency. Visual representations like t-SNE scatter plots illustrate the latent space’s structure, while line and bar plots display training and validation losses. |
| 37 | Jun Wang et al. 2020 | Prediction and Prioritization of Autism-Associated Long Non-Coding RNAs Using Gene Expression and Sequence Features | BrainSpan Atlas of the Developing Human Brain; RNA transcript sequences from GENCODE; 604 high-confidence ASD risk genes and 1,594 non-ASD disease genes. | The BrainSpan dataset provides gene expression profiles from 524 post-mortem brain samples, covering developmental time points ranging from 8 weeks post-conception to 40 years of age. The GENCODE dataset includes RNA transcript sequences, from which the study extracts k-Mer compositions (sequences of k nucleotides) to derive features for prediction. The positive instances in the training dataset consist of 604 genes linked to ASD, while 1,594 genes unrelated to ASD serve as negative controls. | ROC AUC and PR AUC are the primary metrics used for evaluating model performance. Accuracy, sensitivity, specificity, and Matthews Correlation Coefficient (MCC) are additional performance metrics used for model assessment. | The models used are logistic regression (LR), support vector machine (SVM), and random forest (RF) for predicting ASD-associated long non-coding RNAs (lncRNAs), with an autoencoder for dimensionality reduction. | The feature extraction from gene expression data involves applying an autoencoder to reduce high-dimensional data into lower-dimensional representations.  For RNA transcript sequences, the study calculates k-Mer frequencies (k = 1, 2, 3, 4) and applies feature selection using random forest to reduce the number of k-Mer features.  Both gene expression and k-Mer features are then combined for training machine learning models. | This study evaluates model performance using metrics like **ROC-AUC, PR-AUC, accuracy, sensitivity, specificity,** and **Matthews Correlation Coefficient (MCC).** The authors use ten-fold cross-validation to validate the model's performance, utilizing ROC and PR curves for model comparison. Figures illustrate autoencoder performance, k-Mer selection, and model validation through percentile ranking. |
| 38 | Yunxia Tang et al. 2020 | TruNeo: An Integrated Pipeline Improves Personalized True Tumor Neoantigen Identification | Lung cancer dataset (WES and RNA-seq data from paired tumor and normal samples); 1599 single-nucleotide variants (SNVs). | The lung cancer dataset involves WES and RNA-seq from a patient with advanced squamous cell carcinoma, containing 451 somatic mutations, including missense mutations and insertions/deletions (InDels). This focuses on tumor-specific neoantigens formed by somatic mutations in the tumor samples, which are potential targets for immunotherapy. | Key evaluation metrics for neoantigen prediction include recall rates for the top 5, 10, and 20 predictions, IC50 for peptide-MHC binding affinities, and validation through T-cell receptor sequencing and Elispot assays. | The model is TruNeo, a multi-step pipeline that predicts and ranks neoantigens by integrating somatic mutation identification, peptide-MHC binding predictions from NetMHCpan, and a deep learning model that refines rankings using additional biological factors. | Somatic variants (SNVs and InDels) are detected from WES data, and gene expression levels are quantified from RNA-seq data.  Neoantigen prediction involves scanning for MHC class I binding peptides derived from somatic mutations.  The deep learning model uses one-hot encoding for peptide sequences and HLA genotypes and ranks neoantigens based on a combination of binding affinity and expression data. | The study uses **recall rate** and **positive rate** to evaluate TruNeo’s ability to rank immunogenic neoantigens effectively compared to other models. The paper includes bar plots to compare TruNeo’s prediction performance to tools like NetMHCpan, MHCflurry, PSSMHCpan, and DeepHLA, showcasing TruNeo’s superior accuracy in neoantigen ranking. Figures also illustrate the TruNeo pipeline and workflows from sequencing to neoantigen prediction and validation. No F1 score or efficiency metric is reported. |
| 39 | Anand Ramachandran et al. 2021 | HELLO: Improved Neural Network Architectures and Methodologies for Small Variant Calling | Genome-in-a-Bottle (GIAB) repositor, whole-genome sequencing (WGS) data for HG003 and HG001 genomes with multiple sequencing coverages from Illumina, PacBio, and hybrid platforms. | The GIAB dataset contains short-read (Illumina) and long-read (PacBio) data, which are used to perform variant calling, focusing on identifying small variants (1–50 bp). The datasets include coverage-based subsets and use haplotagging for long-read data to improve accuracy in variant calling. | Precision, Recall, and F1-Score are the primary performance metrics used to evaluate small variant calling accuracy. the number of indel errors and substitution errors are compared across different sequencing platforms and coverage points. | The model is HELLO (Hybrid and Stand-alone Estimation of Small Genomic Variants), a deep neural network optimized for small variant calling, which models read-allele relationships to enhance performance in low-coverage settings, compared against the DeepVariant baseline. | Data preprocessing includes alignment of sequencing reads to the reference genome, indel realignment, and haplotagging for long-read PacBio data. The input features are designed to be specific to variant calling tasks, where sequencing reads and their relationships with alleles are used to generate predictions, without converting the problem into an image recognition task like in DeepVariant. | This article presents the **HELLO** model, which is a deep neural network-based method for small variant calling. To showcase output performance, the authors used several diagrams and tables comparing **precision, recall, F1 scores, and error rates** across different sequencing technologies (Illumina, PacBio, and hybrid). Figures demonstrate comparative analysis against **DeepVariant and GATK** tools using **excess error plots** and **performance tables for SNVs and indels.** |
| 40 | Zexian Zeng et al. 2021 | Deep Learning for Cancer Type Classification and Driver Gene Identification | From TCGA database WES data samples across seven cancer types: brain, breast, colorectal, kidney, lung, prostate, and uterus. | Germline and somatic mutations were identified from matched blood and tumor samples, including high- and moderate-impact variants like missense, nonsense mutations, and InDels, along with transcript sequences from 985 canonical RefSeq transcripts. | Precision, recall, and F-measure were key metrics for evaluating the DeepCues model, which achieved 77.6% accuracy in classifying cancer types using combined germline and somatic mutations. | The model is DeepCues, a deep learning-based CNN that classifies cancer types and identifies relevant genes from raw WES data by encoding germline and somatic mutations, outperforming baseline models like logistic regression and SVM. | One-hot encoding was used to represent the DNA sequences, translating each codon into a 64-dimensional binary vector. This encoding was applied to both germline and somatic mutation sequences. The model automatically learns features from these encoded sequences without manual feature engineering. The final output layer classifies the cancer type based on the learned features. | The article uses accuracy and F1 scores to compare the DeepCues model with baseline models, presents precision, recall, and confusion matrices for classification effectiveness, and includes figures showing the model architecture, data processing, and comparative accuracy across cancer types based on germline and somatic mutations. |
| 41 | Yunus Emre Cebeci et al. 2024 | Improving Somatic Exome Sequencing Performance by Biological Replicates | Sequencing Quality Control Phase 2 (SEQC2) consortium dataset; whole exome sequencing (WES) data for tumor and normal samples from the HCC1395 breast cancer cell line. | Tumor and normal samples were sequenced at six different centers, with three replicates from Fudan University, Illumina, and Novartis, and one replicates each from the other three centers.  Data includes variant calls made using Mutect2, Strelka2, and SomaticSniper pipelines, and variants are evaluated using high-confidence regions from the SEQC2 dataset. | Evaluation metrics include Precision, Recall, and F1 score, which measure the accuracy of somatic variant detection across different replicate-based strategies.  The study compares results from within-center, cross-center, and all-center replicate combinations and applies machine learning models using these results. | The model used is NeuSomatic in ensemble mode, trained on variants detected in two or more biological replicates for improved somatic variant detection in WES data. | The study uses common preprocessing techniques for sequencing data, including read trimming (with Trimmomatic), mapping (using bwa and bowtie2), and variant calling (with Mutect2, Strelka2, and SomaticSniper).  Multiple detection variants from replicates are used to train ML models. The models are validated by dividing the dataset into training and test sets using different chromosome regions to avoid overfitting. | The article presents F1 scores, precision, and recall to show accuracy improvements in somatic variant calling using replicate-based consensus approaches, accompanied by diagrams illustrating read counts and mapping statistics, and compares NeuSomatic ensemble model results with high-confidence variant labels. |
| 42 | Mehmet Arif Ergun et al. 2024 | COSAP: Comparative Sequencing Analysis Platform | Whole Exome Sequencing (WES) data from the Sequencing Quality Control 2 (SEQC2) project. | The dataset used in the study is WES data from SEQC2 with accession numbers SRR7890850 and SRR7890851. The SEQC2 project aims to establish community reference samples, data, and call sets for benchmarking cancer mutation detection. | Number of variant callers used: 11  Algorithms included both somatic and germline variant callers.  The pipelines were executed with parallelization and optimized for performance with hardware like GPU and NVMe drives. | The model is COSAP an open-source tool that provides flexible sequencing analysis pipelines, incorporating various tools for preprocessing, mapping, alignment, variant calling, and annotation, allowing users to create custom pipelines for comparative analysis. | Preprocessing: Fastp was used for quality control and filtering of raw reads. BWA and Bowtie2 were used for short-read mapping.  Variant calling and annotation: Tools like Mutect2 and HaplotypeCaller were used for variant calling, while Ensembl VEP and SnpEff were employed for variant annotation. | The article introduces COSAP, a web-based platform for comparative sequencing analysis. Key output metrics and visualizations include: Venn Diagrams and Jaccard Similarities: These illustrate overlaps in variant calls between different sequencing algorithms. Precision and Recall Plots: Shown when ground truth data is available, providing insights into pipeline performance. Performance Comparison Charts: Highlighting runtime improvements across different storage configurations. |
| 43 | Hyunjung Lee et al. 2024 | Assessing the Reliability of Point Mutation as Data Augmentation for Deep Learning with Genomic Data | CCDS (Consensus Coding Sequence), Chromosome-21, Gao15, NN269, and Arabidopsis datasets. | The datasets include CCDS and Chromosome-21 with heavily skewed label distributions (1/25 and 1/4913 ratios), Gao15 with 203 bp sequences from HEK293 cells, NN269 focusing on splice sites from 269 human genes, and Arabidopsis containing 402 bp sequences for acceptor and donor splice sites. | The study involves silent, missense, nonsense, and random mutations in non-coding regions, with up to 10-point mutations per sequence, and evaluates performance using metrics like false-positive rate (fpr80), auROC, auPRC, and precision at 95% recall (Pr95). | The models used are TISRover for translation initiation site detection and SpliceRover for splice site detection, both employing CNNs to analyze the effects of point mutations as a data augmentation technique. | Data preprocessing: Sequences are extracted based on specific genomic characteristics like start codon (ATG) for TIS datasets and splice acceptor/donor sites.  The study employs moderate point mutation as an augmentation strategy to create new data samples without altering essential biological functions. | The study evaluates the effectiveness of point mutations as a data augmentation method for training deep learning models on genomic data, using F1 Score, auROC, and auPRC as key performance metrics, with tables displaying accuracy and figures illustrating changes in neural network performance across mutation types. |
| 44 | Yu-Chiao Chiu et al. 2018 | Predicting drug response of tumors from integrated genomic profiles by deep neural networks | CCLE includes 622 cell lines with genomic profiles and drug response data for 265 drugs, while TCGA provides 9,059 tumor samples for testing across 33 cancer types, featuring genomic data like mutations and expression profiles. | The study leverages high-dimensional mutation and gene expression profiles from CCLE and TCGA, applying these to predict drug responses.  IC50 values for anti-cancer drugs were collected from the Genomics of Drug Sensitivity in Cancer (GDSC) project, representing each drug's effectiveness in reducing cell growth by half. | Model performance was measured primarily by mean squared error (MSE) in IC50 prediction, with optimized model parameters such as neuron count, batch size, and training epochs.  The Adam optimizer and rectified linear unit (ReLU) activation functions were employed in network layers. | The model is DeepDR, a deep neural network that includes a Mutation Encoder pre-trained on TCGA mutation data, an Expression Encoder pre-trained on TCGA expression data, and a Prediction Network that integrates these features to predict IC50 values for anti-cancer drugs, with training also utilizing CCLE data. | Preprocessing: Mutation and gene expression profiles were processed by filtering genes with low variance, encoding mutation data in binary format, and log-transforming expression data.  Feature Selection: Autoencoders were used for dimensionality reduction, and synaptic parameters from pre-trained encoders were used in final model training. | To demonstrate model performance, the article primarily uses:   - MSE evaluates prediction accuracy. - Correlation plots assess IC50 prediction consistency. - Comparative diagrams and density plots compare model performance and IC50 distributions. |
| 45 | Sayed Mohammad Ebrahim Sahraeian et al. 2022 | Achieving Robust Somatic Mutation Detection with Deep Learning Models Derived from Reference Data Sets of a Cancer Sample | **SEQC2 Reference Dataset**: Tumor-normal pairs from HCC1395 (triple-negative breast cancer) and HCC1395BL, with 39,536 somatic SNVs and 2,020 INDELs; includes WGS, WES, and AmpliSeq data from multiple platforms. | The SEQC2 dataset encompasses different sequencing replicates prepared across multiple platforms and sites, with varying coverage levels (10x to 2000x) and tumor purity (5% to 100%) levels, as well as FFPE (formalin-fixed paraffin-embedded) and fresh DNA preparations. | Performance metrics include F1 scores across tumor purity and coverage variations.  Models were evaluated with different tumor/normal purity ratios, read coverages and library preparation methods. | **NeuSomatic**: A CNN-based model for somatic mutation detection, trained on real and synthetic mutations; evaluated against MuTect2, Strelka2, and Octopus across variant types and setups. | Candidate mutations were identified through alignment scanning, with additional preprocessing including read alignment (using BWA-MEM) and deduplication (Picard).  The SEQC2 dataset provided labeled high-confidence and medium-confidence somatic variants, used as ground truth for model training and evaluation. | The article evaluates model performance using the F1 score for somatic mutation detection across varying coverages and tumor purities, heatmaps to illustrate F1 score consistency across datasets, precision-recall analysis for SNVs and INDELs, comparative plots of model performances on WGS, WES, and AmpliSeq, and VAF analysis to assess accuracy across allele frequency distributions. |
| 46 | Alistair S. Dunham et al. 2023 | High-throughput deep learning variant effect prediction with Sequence UNET | The datasets used include **ProteinNet** for structural information and variant frequencies across protein families/species, **ClinVar** for fine-tuning pathogenicity classification of human protein variants, and **DMS & S. cerevisiae** for model validation and generalization. | ProteinNet includes data on protein sequences, structural information, and mutations from over 100,000 PDB structures. The ClinVar dataset contains labeled human variants for pathogenicity.  DMS data and yeast variants validate performance across different species and variant effect predictions. | Trained with hyperparameters optimized for positional scoring matrices (PSSM) and variant frequency classification.  Layers include 6 convolutional layers with 64 filters and a kernel width of 9. Dropout rates were set to 0.05, with batch normalization to improve generalization. | **Sequence UNET**: A CNN with U-shaped architecture for multi-scale sequence representations; supports optional GraphCNN for structural data encoding, fine-tuned for pathogenicity prediction and frequency classification on external datasets. | Protein sequences are one-hot encoded, and structural features are encoded as a graph for the optional GraphCNN module.  PSSMs and variant frequency predictions are calculated from ProteinNet data, with hyperparameter tuning for frequency thresholds to distinguish rare, potentially pathogenic variants. | The article evaluates model performance using the F1 score for accuracy in deleterious mutation classification, ROC and PR curves for precision and recall, Spearman's correlation for ranking variant predictions, heatmaps and comparative plots to compare Sequence UNET with tools like SIFT4G and FoldX, and computation time comparisons to assess scalability. |
| 47 | Raquel Dias et al. 2019 | Artificial Intelligence in Clinical and Genomic Diagnostics | The article covers general AI applications in genomics and clinical diagnostics without focusing on specific datasets. It mentions the utility of datasets like those in variant calling and genome annotation. | Descriptions are high-level, addressing applications in clinical genomics such as variant calling, genome annotation, and phenotype-genotype mapping, rather than detailed data specifics. | Discusses general AI parameters (e.g., input sequences for CNNs in genomics) rather than model-specific hyperparameters. | Various DL methods are used **CNNs** for computer vision tasks and **RNNs** for time series analysis, as well as **transfer learning** and **sequence-to-sequence models** for genomics applications. | AI in genomics tasks often bypasses manual feature extraction due to deep learning’s automatic feature generation. Examples include CNNs for recognizing DNA motifs or RNNs for analyzing sequential DNA data. | The article evaluates AI in genomic diagnostics using ROC curves and F1 scores for variant classification accuracy, heatmaps for visualizing classification task contributions, and comparative tables for comparing deep learning models in clinical genomics, including variant effect prediction. |
| 48 | Yicheng Liu et al. 2024 | MAGPIE: Accurate Pathogenic Prediction for Multiple Variant Types Using Machine Learning Approach | The datasets used **ClinVar, gnomAD** (rare variants for analysis), and **SwissProt & ACMG-guided** datasets for validation. | ClinVar: Contains labeled pathogenic and benign variants for training and validation.  GnomAD: Provides allele frequency information to differentiate rare variants.  SwissProt and ACMG: Serve as orthogonal validation sets to test MAGPIE’s generalization. | MAGPIE uses LightGBM for prediction, employing cross-validation and hyperparameters like tree depth, learning rate, and regularization terms. | MAGPIE uses gradient boosting with feature engineering (AutoFE) and multimodal feature integration to improve prediction across variant types. | Features include functional effects, population-based frequencies, biochemical properties, and conservation scores. Missing values were imputed, and feature importance was assessed. | The article evaluates the MAGPIE model using F1 score for mutation classification balance, ROC and PR curves with AUC scores for performance on balanced and imbalanced datasets, violin and density plots for pathogenic score distributions, and comparative performance charts against other tools across variant types. |
| 49 | Wei Jiao et al. 2020 | A Deep Learning System Accurately Classifies Primary and Metastatic Cancers Using Passenger Mutation Patterns | PCAWG (Pan-Cancer Analysis of Whole Genomes) Dataset and external validation sets | Whole-genome sequencing data from 2,606 tumor samples representing 24 cancer types. The dataset includes mutation profiles, specifically somatic passenger mutations, from primary and metastatic tumors. | Mutation types and regional distribution of somatic mutations; accuracy metrics include F1 scores, recall, and precision. | Deep learning and Random Forest classifiers were used to predict tumor type. The deep learning model achieved high accuracy (91% on PCAWG tumors) by utilizing mutation patterns rather than driver genes. | Mutation features, such as nucleotide substitution types, mutation rate across genomic regions, and potential driver pathways, were used for model input, with preprocessing steps involving the normalization of somatic mutations. | The article evaluates the classifier using the F1 score for accuracy, precision-recall and ROC curves for prediction visualization, heatmaps for tumor type classification accuracy, and confusion matrices for true vs. predicted tumor type distribution. |
| 50 | Hongjian Qi et al. 2021 | MVP predicts the pathogenicity of missense variants by deep learning | HGMD, UniProt, ClinVar: Pathogenic variants (positive samples).  DiscovEHR, UniProt: Non-pathogenic variants (negative samples).  Cancer mutation hotspots, CHD, ASD: Testing data for missense variants. | Large curated pathogenic variant data is used for training, including 32,074 unique positive training variants and 86,620 negative ones.  Cancer mutation hotspot data is also used for performance validation. | Minor allele frequency threshold: MAF < 10−410^ {-4}10−4 for both training and testing data.  For constrained genes (those intolerant to loss of function), ExAC pLI ≥ 0.5; for non-constrained genes, ExAC pLI < 0.5. | The paper introduces MVP (Missense Variant Pathogenicity), a deep learning model using a residual neural network (ResNet) for pathogenicity prediction.  Two distinct models for constrained and non-constrained genes are developed to handle gene dosage sensitivity differences. | Features include local context, amino acid constraints, conservation scores, protein structure and interaction, gene mutation intolerance, and scores from other published prediction methods.  The training utilizes correlation-ordered features through convolutional layers for spatial pattern extraction, with two residual blocks in the ResNet model. | The MVP model’s performance is evaluated using ROC curves for sensitivity and specificity, AUC for performance across constrained/non-constrained genes, precision-recall curves for de novo missense variants, and feature contribution analysis through AUC reduction to identify key accuracy-driving factors. |
| 51 | Berk Mandiracioglu et al. 2024 | ECOLE: Learning to call copy number variants on whole exome sequencing data | 1000 Genomes Project: WES and WGS data.  NA12878, Chaisson et al., Guo et al.: Bladder cancer samples and human expert-curated calls for validation. | The main dataset includes 707 samples (550 for training, 157 for testing) with WES data from Illumina HiSeq 2000 and other platforms and CNV labels created by CNVnator from WGS data. Validation datasets like NA12878 test generalizability, while the bladder cancer dataset fine-tunes somatic CNV calling. | Key parameters include using read depth signal per exon, chromosome-specific classification tokens, and positional encoding to enhance CNV calling accuracy. | ECOLE (Exome-based Copy number variation called LEarner), a deep learning model based on the transformer architecture. ECOLE identifies CNVs by learning from read-depth signals across exons and fine-tuning using limited human expert-labeled samples. | Read-depth data was processed to produce exon-specific embeddings. The model also used positional encoding to capture chromosome context, and fine-tuning was done with expert-labeled data to improve accuracy. | The paper evaluates model performance using the F1 score for overall performance, precision-recall curve in supplementary materials, confusion matrices for CNV type evaluation, and figures of the ECOLE transformer-based architecture and chromosome-wise CNV stratification to visualize system performance. |
| 52 | Haitham A. Elmarakeby et al. 2021 | Biologically informed deep neural network for prostate cancer discovery | Whole-exome sequencing (WES) and RNA-seq data for 1,013 prostate cancer patients. | This dataset includes genomic data from 333 castration-resistant prostate cancers (CRPCs) and 680 primary cancers. Data were used to predict the state of prostate cancer (primary or metastatic) based on somatic mutation and copy number alteration data. | Parameters included mutational features, gene-level copy number variations (amplifications and deletions), and pathway-level features derived from curated pathways in the Reactome database. | **P-NET:** A biologically informed neural network with a hierarchical structure reflecting genes, pathways, and biological processes, constrained by parent-child relationships. | Features include somatic mutations, gene copy number changes, and pathway information derived from Reactome. The preprocessing steps involved aggregating mutation data and applying GISTIC2.0 for copy number variations. | The paper evaluates the model using the F1 score and AUPRC for effectiveness, ROC curve and AUC for accuracy, survival curves for clinical relevance in biochemical recurrence prediction, and a network architecture diagram to visualize the P-NET model structure. |
| 53 | Jian Zhou et al. 2019 | Whole-genome deep-learning analysis identifies the contribution of noncoding mutations to autism risk | Whole-genome sequencing (WGS) data from 1,790 families, part of the Simons Simplex Collection (SSC), focusing on autism spectrum disorder (ASD). | The dataset includes 7,097 genomes, encompassing ASD probands, unaffected siblings, and parents. It captures noncoding de novo mutations and their regulatory effects, specifically examining their impact on transcriptional and post-transcriptional regulation. | The parameters analyzed include transcriptional and post-transcriptional regulatory features. The focus was on transcription factor binding, histone marks, and RNA-binding protein (RBP) profiles to understand the functional impact of noncoding mutations. | **DeepMut**: A CNN-based framework for predicting the functional impact of noncoding mutations on transcriptional and post-transcriptional regulation, trained on biochemical data for single-nucleotide resolution impact predictions. | Biochemical feature extraction included genome-wide transcriptional and RBP interaction profiles. Training data involved annotated functional data from ENCODE and Roadmap Epigenomics, capturing histone marks, chromatin accessibility, and RBP binding sites. | The article evaluates model performance using Disease Impact Score (DIS) for noncoding mutation significance, F1 score and AUC for prediction accuracy, t-SNE clustering plot for ASD risk mutation effects, and box plots and survival curves to visualize noncoding mutation impacts on gene regulation. |
| 54 | Yingshuai Sunet et al. 2019 | Identification of 12 cancer types through genome deep learning | WES data from 6,083 cancer samples across 12 cancer types from TCGA and 1,991 healthy samples from the 1000 Genomes Project. | The dataset includes somatic mutation profiles of various cancer types, including breast, lung, and colorectal cancers, with specific models developed for each type. | The model parameters included optimized layers in the deep learning model with the ReLU activation function, L2 regularization, and exponential decay for learning rate adjustments. | **GDL (Genomic Deep Learning):** A DNN-based model for cancer detection, using multiple hidden layers and SoftMax regression for classification, with specific and mixture models developed to classify cancer types. | WES data was preprocessed to extract point mutations, which were then encoded into binary vectors for model training. Variant files were filtered to include mutation hotspots, and high-frequency sites were selected for input. | The paper evaluates model performance using accuracy, sensitivity, and specificity for cancer type identification, ROC curves and AUC for cancer-specific model classification, confusion matrix for the mixture model's predictions, and cancer stage analysis, achieving a mean accuracy of 97%. |
| 55 | Xiguo Yuan et al. 2020 | STIC: Predicting Single Nucleotide Variants and Tumor Purity in Cancer Genome | Simulation datasets were created with SInC software based on chromosome 21, as well as a real sequencing sample from the European Genome-phenome Archive (EGA) with access number EGAD00001000082. | The simulation datasets included 100,000 somatic SNVs and 50,000 germline SNVs with varying levels of tumor purity, simulated by mixing tumor and normal genomes. The real dataset involved a whole exome sequence from a breast cancer patient, paired with a matched normal sample for certain evaluations. | Key parameters included the AF (allele frequency) thresholds of 0.3 and 0.9 for distinguishing somatic and germline SNVs. Sensitivity, precision, and F1-score were also measured to evaluate performance. | **STIC**: A BP neural network method for distinguishing somatic from germline SNVs based on AF levels and estimating tumor purity by analyzing somatic AF modes and purity relationships. | A set of 19 features related to SNVs (e.g., read count, mapping quality) was extracted from the aligned reads. The reads were aligned using the BWA algorithm, followed by feature extraction to train the neural network for SNV predictions. | The article evaluates the STIC model using sensitivity, precision, and F1 score, ROC curves for accuracy, simulation studies and real sequencing applications for somatic SNV prediction and tumor purity estimation, and overlap density score (ODS) for reliability compared to other methods. |
| 56 | Sina Abdollahi et al. 2021 | WinBinVec: Cancer-Associated Protein-Protein Interaction Extraction and Identification of 20 Various Cancer Types and Metastasis Using Different Deep Learning Models | TCGA Whole-Exome Sequencing (WES) dataset with 9708 samples, and NCKUH Whole-Genome Sequencing (WGS) dataset with 173 samples. | The TCGA dataset covers 20 cancer types with samples at different stages, while the NCKUH dataset includes samples from colorectal, ovarian, and endometrial cancers. | Key evaluation metrics include accuracy, ROC AUC scores, and nDCG values, particularly for cancer-type prediction and metastasis classification. | **WinBinVec**: A custom deep learning model for cancer type classification, using Protein-Protein Interactions (PPIs) and a CNN to analyze mutation regions, compared with binding affinity, self-attention, and GCN-based methods. | Feature extraction methods included the use of binary vectors, gene expression values, biophysicochemical properties, and binding affinity changes upon mutation. WinBinVec segments amino acid sequences into mutation-accumulated windows for optimized feature extraction. | Accuracy: Evaluated through a 10-fold cross-validation.  ROC and PR Curves: Used for performance visualization across models.  Kaplan-Meier Curves: Used for survival analysis related to PPI essentiality.  NDCG Scores: To assess model predictions against MEDICI's PPI essentiality rankings.  Confusion Matrix: To display classification results. |
| 57 | Christos M. Dimitrakopoulos et al. 2017 | Computational Approaches for the Identification of Cancer Genes and Pathways | The multi dataset used in this  TCGA  International Cancer Genome Consortium (ICGC) | It discusses high-throughput sequencing data, including whole-exome sequencing (WES) and whole-genome sequencing (WGS), with a focus on identifying cancer-related somatic mutations and pathways. | Key parameters include sensitivity, specificity, and measures of network connectivity and modularity in cancer pathway predictions. | **Computational Methods for Cancer Gene Identification**: Includes pathway-based, network-based (protein-protein interactions and signaling networks), and de novo methods (detecting co-occurrence and mutual exclusivity of mutations). | Techniques discussed include pathway enrichment analysis, network module detection, and statistical measures for identifying mutually exclusive or co-occurring mutations across genes. | The study explores different computational approaches for identifying cancer pathways, including network-based and mutation pattern detection methods. It uses diagrams and flowcharts to visualize mutations, pathways, and methods. Tables summarize the methods and their categories but don’t provide specific performance metrics like F1 scores or accuracy. The focus is on classifying methods by their application to detect driver mutations in cancer pathways rather than evaluating specific model outcomes. |
| 58 | Ahmad A. Alzahrani et al. 2024 | Machine Learning Approaches for Advanced Detection of Rare Genetic Disorders in Whole-Genome Sequencing | The SweGen dataset from the Swedish Genomics Database. | This dataset contains WGS data from a large cohort of Swedish individuals. It includes comprehensive genetic variations, such as single nucleotide polymorphisms, insertions, deletions, copy number variations, and structural variants. Key attributes encompass demographic data, variant frequencies, functional annotations, and quality metrics. | The study evaluates accuracy, precision, recall, F1-score, and ROC-AUC for model performance. The Random Forest algorithm achieved an accuracy rate of 97%. | The study employs a Random Forest (RF) classification model to identify rare genetic disorders. The RF model handles non-linear gene interactions and large datasets, identifying critical genetic variations for disease prediction. | Data preprocessing includes cleaning, normalization, handling imbalanced data, dimensionality reduction, and splitting the data into training and testing sets. Important genetic features were extracted and ranked based on their relevance to rare disorders, with Synthetic Minority Over-sampling Technique (SMOTE) applied to balance the dataset. | The article presents a flow diagram outlining data preprocessing and classification, an ROC curve for true-positive and false-positive rates, bar plots and feature importance charts for visualizing significant genetic markers, and performance metrics including 97% accuracy, 95% precision, 96% recall, and 97% F1 score. |
| 59 | Neringa Jurenaite et al. 2024 | SetQuence & SetOmic: Deep Set Transformers for Whole Genome and Exome Tumour Analysis | The study utilizes TCGA and COSMIC (Catalogue of Somatic Mutations in Cancer) datasets. | The TCGA dataset includes WES data with somatic variants, primarily focusing on coding regions across 32 tumor types. The COSMIC dataset provides larger-scale WGS data, including non-coding variants, and allows for more comprehensive analysis across tumor types. | Key evaluation metrics include accuracy, precision, recall, and ROC-AUC for tumor type classification. The study also optimizes the SetQuence architecture for computational efficiency, handling large genomic datasets with high-dimensionality features. | SetQuence: A Set Transformer-based model for analyzing variant-associated sequences in WES and WGS data using attention mechanisms.  SetOmic: An extension of SetQuence that integrates multi-omics data for flexible genomic and transcriptomic analysis. | The study employs DNABERT as a feature extraction model for encoding sequences, followed by pooling strategies (e.g., max-pooling, mean-pooling) to create fixed-dimensional representations. Additional preprocessing includes shuffling and freezing sequence sets to optimize memory and computational efficiency during training. | The article includes model architecture diagrams for SetQuence and SetOmic, a confusion matrix for classification accuracy across tumor types, ROC and PR curves for model performance evaluation, a t-SNE plot for tumor type clustering, and performance metrics: 60.5% precision, 51.4% recall, 59.2% F1 score, 70.9% accuracy, and 91% ROC AUC. |
| 60 | Michael Menzel et al. 2024 | Benchmarking Whole Exome Sequencing in the German Network for Personalized Medicine | The study used WES data from six formalin-fixed paraffin-embedded (FFPE) tissue specimens of various cancers and four commercial reference samples. | The dataset consists of matched tumor-normal DNA samples representing diverse cancer types and genomic characteristics, including samples with low and high tumor mutational burden (TMB) and homologous recombination deficiency (HRD). These were processed across 21 German centers to evaluate reproducibility in clinical diagnostics. | Evaluation metrics include positive percentage agreement (PPA), positive predictive value (PPV), concordance for variant calls, copy-number alterations (CNAs), and complex biomarkers (TMB, HRD, and MSI). | This study benchmarked local wet-lab and bioinformatic workflows at each center, followed by a central bioinformatic pipeline to separate wet- and dry-lab variability. Different variant filtering criteria and sequencing depths were analyzed to assess the impact on accuracy. | Data preprocessing involved DNA extraction, sequencing, and filtering of variant calls (e.g., depth, variant allele frequency), followed by re-analysis with a centralized pipeline to enhance standardization and reduce discrepancies. | The article presents a confusion matrix for variant call discrepancies, ROC curves for accuracy assessment in HRD, TMB, and MSI across centers, bar and line graphs for variant distribution and copy number concordance, and performance metrics including 89% PPV, 76% PPA (increased to 88% with standardization), and 93-94% concordance rates for complex biomarkers like HRD, TMB, and MSI. |
| 61 | Nandini G. Sandran et al 2024 | Application of multiple mosaic callers improves post-zygotic mutation detection from exome sequencing data | Australian Cerebral Palsy Biobank (ACPB): WES data from 145 parent-child trios. Simons Simplex Collection (SSC): WES data from 405 families. | The datasets include exome sequences of children with neurodevelopmental disorders and their parents. The ACPB focuses on cerebral palsy cases, while the SSC includes autism spectrum disorder cases. The analysis identified mosaic variants in probands and their parents, assessing the prevalence of post-zygotic mutations. | Evaluation metrics include validation rates for variant detection, with high-depth sequencing used for confirmatory testing. Validation rates reached 69.2% for M3trio (for child variants) and 92.7% for pGoM (parental mosaic variants). | **M3 & pGoM**: Detection pipelines for child somatic mosaicism (M3) and parental mosaicism (pGoM), using mosaic variant callers like Mutect2, MosaicHunter, and MosaicForecast to prioritize high-confidence variants confirmed by at least two callers. | Data preprocessing included mapping reads to the genome, followed by variant filtering based on alternate allele frequency (AAF) thresholds, and minimum read support. Variants were prioritized using criteria from in silico prediction tools such as CADD, Polyphen, and SIFT. | The study utilized validation metrics, including high-depth amplicon sequencing and droplet digital PCR (ddPCR) for accuracy.  Validation rates and true-positive rates were reported for detecting mosaic variants in both parent and proband data.  F1 score or efficiency values were not specifically detailed in the article; instead, validation and detection rates were emphasized to measure the effectiveness of the mosaic detection pipeline. |
| 62 | Prashant Gupta et al. 2020 | Deep Learning Discerns Cancer Mutation Exclusivity | Exome sequencing data from the Exome Aggregation Consortium (ExAC) and the Catalogue of Somatic Mutations in Cancer (COSMIC). | The study uses approximately 60,000 exomes from ExAC and additional somatic mutations from COSMIC, focusing on analyzing SNVs on the sex chromosomes, especially Chromosome X. Variants included both cancer-specific and non-cancer SNVs for comparing distribution patterns. | Evaluation was conducted using the Average Precision (AP), with a score of 0.75 for validation data, indicating the model's ability to classify cancer-related mutations. A Mann-Whitney U-test showed statistical significance in distinguishing between cancer and non-cancer mutations. | **Aminoacid Switch Sequence Model (ASSM):** A deep neural network model that converts SNVs into vectorized representations, using bi-LSTM layers and an attention mechanism for classifying variants as cancer or non-cancer, with Skipgram embeddings for mutation context encoding. | ASSM generated 300-dimensional embeddings for each SNV using context from the Skipgram model, a commonly used word-embedding technique. This enabled learning mutation-specific vector spaces, allowing the model to distinguish cancer-related mutations based on context alone. | The study used several diagrams and performance metrics, including a Precision-Recall (PR) curve after 200 training epochs, boxplots showing prediction scores for various mutations (including cancer-specific SNVs), and an average precision (AP) score of 0.75, highlighting the model's effectiveness in identifying cancer-related mutations. |
| 63 | Zexian Zeng et al. 2021 | Deep Learning for Cancer Type Classification and Driver Gene Identification | The study used WES data from TCGA, covering 4,174 samples from seven major cancer types. | This dataset includes exome sequences with germline and somatic mutations (e.g., insertions and deletions) across seven cancer types, including breast, lung, colorectal, brain, kidney, prostate, and uterine cancers. Both germline and tumor DNA sequences were included to investigate cancer-specific genetic variations. | Performance metrics included overall classification accuracy, precision, recall, and F-measures. The model achieved an accuracy of 77.6% using cancer sequence data and 73.9% with only germline sequences. | **DeepCues**: A deep learning model using CNNs for cancer type classification and gene identification, leveraging raw DNA sequences and both germline and somatic mutation data. | Data preprocessing involved one-hot encoding of germline and somatic mutations and the integration of mutation data into a unified sequence format. CNN layers automatically derived features from these raw encoded sequences without manual feature engineering. | F-measure: Used for performance evaluation across cancer types.  Precision and Recall: Provided for each cancer type, allowing a detailed assessment of classification performance.  Accuracy Comparisons: Illustrated through bar charts comparing DeepCues model with conventional methods (logistic regression, SVM). Confusion Matrix: Showcased the prediction performance for different cancer types. |
| 64 | C. Sateesh Kumar Reddy et al. 2023 | Deep Learning Framework for Cancer Type Classification Using Genome Sequencing with AlexNet | TCGA dataset | The dataset includes genome sequencing data from various cancer types, primarily focusing on tumor samples. TCGA provides a wide range of molecular data types, with this study using specific genomic features across multiple cancers, such as lung, ovarian, and glioblastoma. | Performance metrics include accuracy, sensitivity, specificity, precision, and F1-score. The proposed model achieved an accuracy of 98.54%, outperforming previous models such as SVM and CNN-based classifiers. | **AlexNet-based Model**: A deep learning model for multi-class cancer type classification, using Ada-boost feature extraction to identify deep features highlighting inter- and intra-cancer relationships. | Ada-boost was used as a feature extraction method to capture specific genomic patterns. The extracted features were processed through AlexNet’s layers, which included convolution, pooling, and fully connected layers to perform multi-class classification. | F1-score: Demonstrated superior performance with an F1-score of 98.76%.  Accuracy, Sensitivity, Specificity, and Precision: These metrics were used to evaluate model performance, with the proposed method showing 98.54% accuracy, 97.76% sensitivity, 98.31% specificity, and 99.48% precision.  Graphical Comparison: Performance metrics were compared graphically against other models (e.g., SVM, CNN, and DLCNN) to demonstrate the effectiveness of the AlexNet model in cancer classification. |
| 65 | Weisheng Zheng et al. 2023 | Deep learning model accurately classifies metastatic tumors from primary tumors based on mutational signatures | Whole Exome Sequencing (WES) data from The Cancer Genome Atlas (TCGA) and several other metastatic tumor cohorts. | This dataset includes over 9,700 primary tumors from more than 25 cancer types and about 1,500 metastatic tumors across over 30 cancer types. The data covers somatic mutations and mutational signatures that distinguish primary from metastatic tumor samples. | Performance metrics for the model include accuracy, F1 score, recall, Area Under the Curve (AUC), and Average Precision (AUPRC). MetaWise achieved an accuracy of 88.5% and an AUPRC of 90%. | **MetaWise:** A deep neural network model that classifies primary and metastatic tumors using SBS, DBS, and small insertion/deletion mutational signatures, interpreted with SHAP and LIME to identify influential signatures. | Somatic mutations are characterized by mutational signatures calculated using COSMIC references. SBS, DBS, and ID mutational signatures are derived and used as model inputs. SHAP and LIME analyses are applied to understand signature importance in classification. | The model effectiveness using F1 score, recall, AUC, and AUPRC for classifying metastatic vs. primary tumors, with a performance comparison table against traditional ML models (SVM, Random Forest) and DiaDeL; SHAP and LIME visualizations highlight mutational signature contributions, focusing on DNA repair deficiencies and APOBEC mutations, while DRF analysis shows mutational signature distribution differences between primary and metastatic cancers. |
| 66 | Yingshuai Sun et al. 2019 | Identification of 12 Cancer Types Through Genome Deep Learning | WES data from TCGA and the 1000 Genomes Project. | The dataset includes WES data of 6,083 samples across 12 cancer types (e.g., breast, bladder, lung, and ovarian cancers) from TCGA and 1,991 healthy samples from the 1000 Genomes Project. This data was used to identify genomic patterns associated with each cancer type and distinguish between cancerous and non-cancerous samples. | The models were evaluated based on accuracy, sensitivity, and specificity. The cancer-specific models achieved accuracies between 97.47% and 100%, while the mixture model had an accuracy of 70.08% for distinguishing between all 12 cancer types. | **Genomic Deep Learning (GDL):** A deep neural network approach using TensorFlow for cancer classification, with cancer-specific, total-specific, and mixture models, optimized with L2 regularization and exponential decay learning rate, and a SoftMax output layer. | Data preprocessing included selecting high-frequency mutation sites from the cancer samples to optimize computational efficiency, reducing redundancy. Variants from cancer and healthy samples were encoded using one-hot encoding. For the DNN, each cancer type was transformed into a unique array for effective classification. | Accuracy, Sensitivity, and Specificity: These metrics are reported for multiple models, achieving accuracy as high as 97.47% for specific cancer models and 70.08% for a mixed model across 12 cancer types.  ROC Curves and AUC: Displayed to show the performance of the models, with AUC scores over 96% for most cancer types.  Confusion Matrix: Used for the mixed model to illustrate prediction accuracy across different cancer types.  Comparison Tables: Provided for accuracy, sensitivity, and specificity across various models. |
| 67 | Prima Sanjaya et al. 2023 | Mutation-Attention (MuAt): deep representation learning of somatic mutations for Tumour typing and subtyping | Pan-Cancer Analysis of Whole Genomes (PCAWG): 2,587 whole genomes across 24 tumor types.  TCGA: Exome sequencing data with 7,352 exomes covering 20 tumor types. | The datasets include somatic mutations from whole-genome sequencing (WGS) and whole-exome sequencing (WES) for various cancer types. The mutations include single-nucleotide variants (SNVs), multi-nucleotide variants (MNVs), small insertions and deletions (indels), and structural variants (SVs). | Performance was evaluated based on accuracy, with MuAt achieving 89% accuracy for WGS and 64% for WES, as well as top-5 accuracy metrics (97% for WGS and 90% for WES). | **MuAt:** A deep neural network (DNN) with an attention mechanism to focus on individual mutations, processing mutation types, genomic positions, and annotations for tumor classification. | Mutation types and genomic positions are encoded in one-hot representations, with sequence motifs and positional bins used to represent the data. The model integrates these features with attention layers to improve interpretability and classification accuracy. | The paper reports classification performance using accuracy, precision, recall, and F1 scores, along with Top-1, Top-3, and Top-5 accuracy metrics, visualizes feature space and tumor clustering with UMAP plots, and uses confusion and attention matrices to show prediction accuracy and learned patterns in mutation types across tumor types. |
| 68 | Weisheng Zheng et al. 2023 | Deep Learning Model Accurately Classifies Metastatic Tumors from Primary Tumors Based on Mutational Signatures | Whole-exome sequencing (WES) data from TCGA and other metastatic cohorts, including 9,000 primary and 1,500 metastatic tumor samples. | The dataset consists of somatic mutations from primary and metastatic tumors across over 25 cancer types, focusing on single-base substitutions (SBS), double-base substitutions (DBS), and small insertions/deletions (IDs). | Key metrics include accuracy (88.5% for metastatic tumor identification), F1 score, recall, AUC, and AUPRC. | **MetaWise:** A fully connected deep neural network model for classifying primary and metastatic tumors, using mutational signatures from SBS, DBS, and IDs, with SHAP and LIME for model interpretation. | Features are derived from mutational signatures calculated through COSMIC, with additional filtering based on frequency thresholds (2.5%, 5%, or 10%) to remove noise. Non-coding mutations were also incorporated to improve classification accuracy. | The article presents model evaluation using F1 score, recall, AUC, and AUPRC, with confusion matrices for classification accuracy, and SHAP and LIME analyses for feature importance and model interpretability. |
| 69 | Zexian Zeng et al. 2020 | Deep Learning for Cancer Type Classification and Driver Gene Identification | TCGA Dataset | The study uses WES data for 4,174 cancer samples across seven major cancer types (breast, colorectal, brain, uterus, lung, kidney, and prostate). Both germline variants and somatic mutations are analyzed. | Performance metrics include accuracy, precision, recall, F-measure, and AUC for classifying cancer types and identifying relevant genes. | **DeepCues**: A CNN-based deep learning model that uses raw WES data for cancer type prediction and relevant gene identification. | Raw DNA sequences are transformed using one-hot encoding for each codon, resulting in a 64-dimensional vector per codon. Germline and somatic mutations are combined for feature representation, and functional annotation is applied to classify mutations by their effects. | The paper evaluates model performance using accuracy, precision, recall, and F1 scores, with confusion matrices for cancer type classification, and includes performance comparison charts showing CNN accuracy against traditional models. |
| 70 | Firda Aminy Maruf et al. 2021 | DNN-Boost: Somatic Mutation Identification of Tumor-Only Whole-Exome Sequencing Data Using Deep Neural Network and XGBoost | Tumor-only WES dataset of pancreatic cancer obtained from the NCBI Sequence Read Archive (SRA). | The dataset included 6 paired tumor-normal samples in FASTQ format, and additional tumor-only WES samples. Variants were called and processed from both paired and tumor-only WES data. | The study’s metrics include accuracy, precision, recall, and F1-score for classifying somatic mutations. | **DNN-Boost:** An ensemble model combining XGBoost for feature selection and a DNN for classification, using variant data processed by Mutect2, GATK HaplotypeCaller, and BCFtools. | Features include variant statistics and functional prediction scores from ANNOVAR. Missing feature values were imputed using k-nearest neighbors, and feature selection was optimized with XGBoost, which reduced noise and improved classification by selecting the top 24 most informative features. | The paper evaluates the model’s classification performance using accuracy, precision, recall, and F1 score, with feature importance plots (XGBoost), a confusion matrix for somatic mutation and germline variant classification, and performance comparison plots contrasting DNN-Boost with methods like Mutect2. |
| 71 | Weisheng Zheng et al. 2022 | Deep Learning Model Accurately Classifies Metastatic Tumors from Primary Tumors Based on Mutational Signatures | TCGA and additional metastatic tumor datasets from cBioPortal. | Whole-exome sequencing (WES) data of more than 9,700 primary tumors across 25 cancer types and over 1,500 metastatic tumors spanning 30 cancer types. | Performance metrics included accuracy, F1-score, precision, and recall. Test accuracies of 86% were achieved with non-coding mutations included. | The **MetaWise** model is a 10-layer fully connected deep neural network (DNN) designed to distinguish primary tumors from metastatic tumors using mutational signatures as input features. | Mutational signatures included single-base substitutions (SBS), double-base substitutions (DBS), and insertion-deletion mutations (ID) derived from both coding and non-coding regions. These signatures were computed using SigProfiler and curated through COSMIC. | The paper evaluates classification performance using F1 Score, Recall, and Test Accuracy, employs SHAP and LIME for feature importance, uses DRF plots for mutational signature differences, and includes heatmaps and comparison tables for model performance across datasets. |
| 72 | Yi-Ching Tang et al. 2018 | Explainable Drug Sensitivity Prediction Through Cancer Pathway Enrichment | Cancer Cell Line Project (CCLP)  Genomics in Drug Sensitivity in Cancer (GDSC) | The datasets include genomic profiles from 787 human cancer cell lines and drug sensitivity data for 244 drugs. It covers extensive profiling of somatic genomic alterations and drug sensitivity across a broad array of cancer types. | The parameters evaluated include genomic mutation positions, mutation status, drug molecular fingerprints, and experimentally measured IC50 values for anticancer compounds. | The study developed a "Cancer Drug Response Profile scan (CDRscan)," a deep learning model using a dual convergence architecture that predicts drug effectiveness based on the genomic signature of cancer cell lines. | Features include genomic mutation status encoded as binary digits and molecular fingerprints of drugs obtained through PaDEL-descriptors. The genomic data encompasses mutation positions and statuses, while the drug data includes molecular structures and properties encoded as binary descriptors. | Mean Absolute Error (MAE)  Root Mean Square Error (RMSE)  Receiver Operating Characteristic (ROC) Curves  Shapley values to visualize feature importance and model explainability  Scatter plots and bar graphs for prediction performance comparisons |
| 73 | Wei Peng et al. 2021 | A Graph Convolution Network-Based Model for Prioritizing Personalized Cancer Driver Genes of Individual Patients | Dataset used form VISDB database (VISs) for Hepatitis B virus (HBV), Human herpesvirus (HPV), and Epstein-Barr virus (EBV). | The VISDB database contains a total of 77,632 VISs of five DNA viruses and four RNA retroviruses. For this study, the datasets included 20,588 VISs for HBV, 5,118 for HPV, and 1,112 for EBV. | The study focuses on identifying and predicting oncogenic viral integration sites in the human genome using features derived from the DNA sequences of these integration sites. | The methodology involves a deep convolutional neural network (CNN) model with attention architecture, named DeepVISP. This model is designed to predict oncogenic virus integration sites by learning informative features and essential genomic positions directly from DNA sequences. | The feature extraction involves encoding DNA sequences into a format suitable for the CNN. The method uses one-hot encoding of nucleotide sequences, where each nucleotide is represented as a 4-dimensional binary vector. This encoding serves as input to the convolutional layers of the neural network, facilitating the automatic learning of features relevant to viral integration sites. | The paper uses metrics such as F1-score, precision, recall, and Mean Reciprocal Rank (MRR) to evaluate the model’s accuracy.  Visual representations include F1-score curves, precision-recall curves, and tables comparing MRR values to baseline methods.  Figures depict model performance across various benchmarks and highlight the gene ranking among personalized cancer driver genes. |
| 74 | Delora Baptist et al. 2024 | Deep Learning for Drug Response Prediction in Cancer" by Delora Baptista | METABRIC (Molecular Taxonomy of Breast Cancer International Consortium) | This dataset contains data from 1,980 primary breast cancer samples including 31 clinical characteristics, mRNA levels (z-scores for 331 genes), and mutation data (175 genes) for a total of 1,904 breast cancer patients. | Various clinical parameters, gene expression data, and mutation details are used to train the deep-learning models. | Multiple deep learning architectures, including (LSTM), Variational Autoencoders (VAEs), and (GCNs), all optimized by Stochastic Gradient Descent (SGD). | The preprocessing includes handling of missing values, data normalization, outlier detection, and feature selection focused on high-risk genes and critical mutations. Data was also scaled and encoded (one-hot and label encoding) for model input preparation. | F1 Scores, AUROC (Area Under Receiver Operating Characteristic Curve), and AUPRC (Area Under Precision-Recall Curve) to evaluate the performance of their predictive models.  Accuracy and MSE (Mean Squared Error) are also included for assessing model quality. |
| 75 | David Earl Hostallero et al. 2021 | Looking at the big picture: incorporating bipartite graphs in drug response prediction | Genomics of Drug Sensitivity in Cancer (GDSC), Cancer Cell Line Encyclopedia (CCLE), Cancer Therapeutics Response Portal (CTRP), and others. | These datasets involve high-throughput screening of various drugs against cancer cell lines, characterized at the molecular level, providing data for drug sensitivity analysis. | The parameters mentioned involve molecular and chemical data used to model the drug response, focusing on understanding complex biological and chemical relationships. | Deep Learning methods, specifically various architectures like DNNs, CNNs, and RNNs, are discussed in terms of their application to drug response prediction. | The article mentions the capability of deep learning to handle large volumes of high-dimensional and noisy data without extensive preprocessing or feature selection. | The study employs AUROC and Spearman’s Correlation Coefficient to evaluate model performance, illustrating the BiG-DRP pipeline and comparing metrics across baseline and graph-based models in figures and tables. |
| 76 | Mira Barshai et al. 2022 | G4detector: Convolutional Neural Network to Predict DNA G-Quadruplexes | Novel datasets based on high-throughput G4 measurements from the **hg19 genome** using the **G4-seq protocol.** | The dataset includes sequences of G-quadruplex (G4) forming structures detected using the G4-seq technique. The sequences were preprocessed to ensure they were of the same length, centered on G4-seq peaks. The study used **two stabilizers**, K+ and K++PDS, which resulted in two sets of sequences representing G4-forming regions in the human genome. Additionally, three types of negative datasets (random, Di shuffled, and PQ predicted negatives) were generated for training and evaluation. | The study introduces **G4detector**, a **CNN** model designed to predict G-quadruplex (G4) forming sequences. | The model is a convolutional neural network that takes one-hot encoded DNA sequences as input, optionally integrates RNA structure information, and predicts G4 structure formation, optimized with the Adam optimizer and a leave-chromosome-out training approach. | DNA sequences were one-hot encoded.  RNA structure information was also incorporated in some models by computing the probability of each nucleotide being unpaired. The study used pybedtools to retrieve G4 sequences and RNAplfold to calculate RNA secondary structure probabilities. | The study uses metrics such as the AUC, precision-recall curves, and accuracy to evaluate performance. The output visualizations include ROC curves, attribution scores, and mutation sensitivity heatmaps to interpret the deep learning model's sensitivity to specific features. |
| 77 | Mengmeng Wu et al. 2016 | Global inference of disease-causing single nucleotide variants from exome sequencing data | 1000 Genomes Project Phase I, exome sequencing data from epileptic encephalopathies and autism spectrum disorders (ASD). | Simulation datasets: Derived from the 1000 Genomes Project, containing known causal variants across exon, promoter, intron, and splice site regions.  Real exome sequencing data: 264 probands for epileptic encephalopathies and 175 ASD cases, which included nonsynonymous de novo mutations. | Mean Rank Ratio (MRR): Used to assess the performance of their method in ranking causal variants.  Area Under the Curve (AUC): A metric used to evaluate the prioritization accuracy of causal variants.  False Positive Rate (FPR) and True Positive Rate (TPR): Used for ROC analysis. | Glints is a novel computational method that prioritizes disease-causing variants by integrating 14 functional scores and 9 association scores using Fisher's combined probability test, validated through simulations and applied to real exome sequencing datasets. | The workflow of Glints involves categorizing variants into four regions: Exon, Promoter, Intron, and Splice site.  It applies a variety of functional prediction scores (CADD, PolyPhen2, SIFT) and integrates them with association scores using a multivariate regression model to predict the likelihood of a variant causing a disease.  The functional scores are converted into p-values, and the final ranking of variants is done through the integration of both gene-level and variant-level information. | The study primarily demonstrates results using metrics like AUC (Area Under the Curve), mean rank ratio (MRR), and "TOP" counts (the number of causal variants ranked in the top 10). Additionally, figures illustrate performance across different populations using ROC-like rank curves, showing Glints' efficiency and comparison with other methods. |
| 78 | Sanghoon Lee et al. 2024 | iGenSig-Rx: an integral genomic signature-based white-box tool for modeling cancer therapeutic responses using multi-omics data | Breast cancer  CALGB 40601, ACOSOG Z1041, NOAH, NSABP B-41. | These datasets provide multi-omics data, including RNA-seq and WXS, for patients treated with Trastuzumab in trials like CALGB 40601 and ACOSOG Z1041, covering genomic features such as mutations and gene expression, with transcriptomic data for all trials and WXS data for select ones. | The model's predictive performance, evaluated using AUROC, achieved 0.89 in CALGB 40601, 0.80 in ACOSOG Z1041, and 0.75 in NOAH and NSABP B-41, while accounting for sequencing errors and integrating multi-omics data. | GenSig-Rx is a white-box tool for modeling cancer therapeutic responses using multi-omics data, optimizing feature weights with Pearson correlations while avoiding overfitting and ensuring stability across clinical trials. | Multi-omics feature extraction: Genomic features were extracted from RNA-seq, Whole Exome Sequencing, and somatic mutation data.  Genomic signatures were calculated using methods like the Otsuka-Ochiai coefficient to assess feature redundancy.  Preprocessing involved removing redundant features through hierarchical clustering and integrating genomic features across multiple datasets for robust modeling. | The article employs AUROC for model performance evaluation, Kaplan-Meier curves for recurrence-free survival, correlation heatmaps for genomic-feature relationships, and pathway analysis diagrams for enriched pathways related to therapy response. |
